# Indicators and Measurement Tools for Health Systems Integration: A Knowledge Synthesis

**DOI:** 10.5334/ijic.3931

**Published:** 2017-11-13

**Authors:** Esther Suter, Nelly D. Oelke, Maria Alice Dias da Silva Lima, Michelle Stiphout, Robert Janke, Regina Rigatto Witt, Cheryl Van Vliet-Brown, Kaela Schill, Mahnoush Rostami, Shelanne Hepp, Arden Birney, Fatima Al-Roubaiai, Giselda Quintana Marques

**Affiliations:** 1 Faculty of Social Work, University of Calgary, CA; 2 Workforce Research and Evaluation, Alberta Health Services (AHS), CA; 3 School of Nursing, University of British Columbia, Okanagan (UBCO), CA; 4 School of Nursing, Universidade Federal do Rio Grande do Sul (UFRGS), BR; 5 University of British Columbia, Okanagan (UBCO), CA; 6 British Columbia Patient Safety and Quality Council (BCPSQC), CA

**Keywords:** Integration, measurement instruments, tools, performance measurement, knowledge synthesis

## Abstract

**Background:**

Despite far reaching support for integrated care, conceptualizing and measuring integrated care remains challenging. This knowledge synthesis aimed to identify indicator domains and tools to measure progress towards integrated care.

**Methods:**

We used an established framework and a Delphi survey with integration experts to identify relevant measurement domains. For each domain, we searched and reviewed the literature for relevant tools.

**Findings:**

From 7,133 abstracts, we retrieved 114 unique tools. We found many quality tools to measure care coordination, patient engagement and team effectiveness/performance. In contrast, there were few tools in the domains of performance measurement and information systems, alignment of organizational goals and resource allocation. The search yielded 12 tools that measure overall integration or three or more indicator domains.

**Discussion:**

Our findings highlight a continued gap in tools to measure foundational components that support integrated care. In the absence of such targeted tools, “overall integration” tools may be useful for a broad assessment of the overall state of a system.

**Conclusions:**

Continued progress towards integrated care depends on our ability to evaluate the success of strategies across different levels and context. This study has identified 114 tools that measure integrated care across 16 domains, supporting efforts towards a unified measurement framework.

## Background

Integrated care is considered a powerful cure for all that ails health systems in most developed economies: poor performance at increasing cost, fragmentation of services, and lack of human resources to care for the aging population [1,2,3]. In their definition, Kodner and Spreeuwenberg [4] comprehend ‘Integrated Care’ as “a coherent set of methods and models on the funding, administrative, organizational, service delivery and clinical levels designed to create connectivity, alignment and collaboration within and between the cure and care sectors” (p.3). In using this broad definition for our review, we surmise that integrated care may refer to the system as a whole or to individual components within the broader health system and we use integrated care synonymously with health systems integration.

Despite far reaching support for integrated care and evidence of promising outcomes [5,6,7,8,9], achieving integrated health systems remains challenging. This has been attributed to ongoing conceptual ambiguity of integrated care and what successful integration looks like in different contexts [2,3,10,11,12]. Continued progress towards integrated care will depend much on our ability to contrast and compare the impact of strategies across different levels and context. However, the complex interplay of structures, processes and outcomes of integrated care is difficult to disentangle, hampering evaluation of progress [13,14]. Besides conceptual ambiguity, measuring integrated care is challenging because of a lack of tools to measure different aspects of integration and inherent difficulties in tracking down existing tools within a dispersed body of literature [15,16]. Being able to measure and evaluate the success of integration strategies in a consistent way is essential to effectively advance the design and implementation of an integrated health system [10].

The aim of this knowledge synthesis was to identify meaningful and relevant integration measurement domains and to search for and select appropriate instruments to measure these domains. The specific research questions were: 1) what are appropriate indicator domains for each of the 10 key integration principles identified in our previous work [17]? and 2) what measurement tools exist to measure these indicator domains?

Our review will contribute to the growing body of literature concerned with measuring progress towards fully integrated systems [2,3,10,18,19,20] and will offer a useful resource to health system planners and decision-makers.

### Theoretical Foundation for our Review

In earlier research, our team synthesized definitions and models for integrated care to encourage consolidation efforts [17]. We found more than 70 definitions and, not surprisingly, no ultimate integration model. We identified, however, ten key principles that cover multiple domains that collectively support integrated care. The key principles are: 1) comprehensive services across the continuum of care, 2) patient focus, 3) geographic coverage and rostering, 4) standardized care delivery through interprofessional teams, 5) performance management, 6) information technology, 7) organizational culture and leadership, 8) physician integration, 9) governance structure, and 10) financial management [17]. Using these key principles, integrated care is conceptualized as ten distinct areas that need to be addressed to successfully create connectivity, alignment and collaboration within and across care sectors. Others have uncovered similar constructs confirming the importance of a range of structural and process elements at different levels to achieve integrated care, collectively advancing the field towards a unified conceptual framework [2,12,21,22].

Our ten key principles have proven useful for decision-makers and service planners for designing integrated care models [23]. However, they are not always easy to measure due to their broad and abstract nature. To advance our previous work, the current systematic review aimed to identify domains and measurement instruments for each of the ten principles. We understand indicator domains to be measurable concepts that capture specific aspects of a key principle. For example, patient engagement would be a measurable indicator domain for the principle of patient focus. We defined measurement instruments as any measurement devices (questionnaires, rating scales, checklists, observation forms) that can be completed by researchers, administrators or participants to measure structures, processes or outcomes associated with an indicator domain such as patient engagement.

By using our key principles as a starting point, we offer a cohesive approach to measuring and evaluating a health system’s state of integration that is grounded in solid research.

## Methods

The knowledge synthesis followed the methods outlined by Levac, Colquhoun & O’Brien [24] and consisted of three components: 1) Delphi process to identify the most relevant indicator domains from the health providers, decision-maker, and researcher perspectives; 2) focus groups with patients to elicit their perspectives on most relevant integration principles; and 3) systematic review of tools for each identified indicator domain. In this study, we report on the Delphi process and the review of measurement tools.

To enhance the global applicability of the work, we developed a partnership with researchers, decision-makers and policy makers in a large urban centre in southern Brazil (Rio Grande do Sul) and Canada (Alberta and British Columbia). Both countries have publicly funded health systems, comparable funding priorities and similar geography of large urban centres and rural communities. Furthermore, health systems integration is a priority in both countries. Brazilian research team members were actively involved in the study from the development of the proposal, data collection and analysis, and interpretation of the data. Guided by integrated knowledge translation principles [25] we engaged knowledge-users (decision-makers and policy-makers) from each jurisdiction throughout the process. The ethics boards of the three participating jurisdictions approved the research protocol.

### Delphi survey

A modified Delphi method [26] was used to reach consensus on the most relevant integration indicator domains. We used the 10 key principles identified in our previous work [17] as starting point. Drawing on the literature, research team members generated a preliminary list of indicator domains for each of the 10 principles. From this list, a Delphi survey was developed in English and translated into Portuguese. We invited 39 integration experts, policy and decision-makers, and health care providers from Canada, Brazil, Europe and the United States to rate the fit and importance and rank priority for each domain. Potential participants were identified by research team members, through the literature, and through research databases of health researchers. Our research coordinator completed an extensive scan of potential panel experts through google searches prior to finalizing the list of participants. The initial survey contained 21 indicator domains across the ten key principles. Participants ranked appropriateness and relevance of each indicator on a scale from 1–5 (1 being most relevant/appropriate). During the first round, participants suggested additional domains, which were included in the second round. The goal was to achieve 75% agreement for inclusion (1 and 2 ratings) or exclusion (4 and 5 ratings) of indicator domains through several survey rounds.

### Searching, selecting and appraising relevant studies

We conducted independent, iterative searches for each indicator domain resulting from the Delphi process within the following broad disciplines: Health Sciences, Education and Management/Business using the core bibliographic databases from these fields (Medline including the Cochrane database of systematic reviews, EMBASE, PsycINFO, CINAHL, ABI Inform, and Business Source Premier). Our research librarian assisted us in identifying search terms specific for each of the indicator domains. We conducted two additional searches for health systems integration and instrument/tool development. The domain specific searches were then combined with the health systems and tool development searches to retrieve relevant articles on measurement tools. We completed an advanced google search to find tools in the grey literature. Results were filtered for date and language and the first 50 documents returned were screened. Research assistants also searched websites of relevant government agencies and research organizations (e.g., Institute for Healthcare Improvement), reference lists of included studies, and citations identified through forward citation searching using Web of Science for relevant tools. The librarian in Brazil used the LILACS database and included abstracts in English and Portuguese.

The research team developed, tested and refined inclusion and exclusion criteria for selecting studies. The key inclusion criteria were: 1) articles must include some kind of instrument to measure structures, processes or outcomes associated with one or more integration domains identified through the Delphi process; 2) instruments must be relevant to the health care context; 3) English and Portuguese articles; and 4) published between 1995 and 2014. In discussion of inclusion and exclusion criteria, a decision was made to exclude articles that focused on administrative data. Administrative data can be influenced by various components within and outside of health systems that may not necessarily be related to integration and were thus beyond the scope of this knowledge synthesis. Instruments that measured integration aspects outside of the identified measurement domains, and instruments primarily focusing on clinical outcomes of integrated care (e.g. patient health outcomes) were also excluded. All research team members involved in rating abstracts participated in training sessions where each individual rated the same 50 abstracts. Results were discussed during meetings, criteria clarified and refined if needed. Further rounds were conducted until the desired level of consistency was achieved. We then assigned two researchers to each indicator domain to read and rate abstracts; disagreements were resolved by a third. We developed and tested a template to guide extraction of relevant information and adapted tools [27] to rate relevancy and quality of articles. The data extraction table was organized around domains and focused, where possible, on the original article that described the instrument development. As a result, if the development article was older than 1995 it was still included. Team members conducted audits for each indicator domain at the relevancy stage and extraction stage to ensure consistency.

Two research team members in Brazil followed the same procedures to complete abstract screening and article selection for English and Portuguese abstracts identified through the Lilacs database. Audits were also conducted as outlined above. Their findings were then integrated into the synthesis.

**Figure 1:**
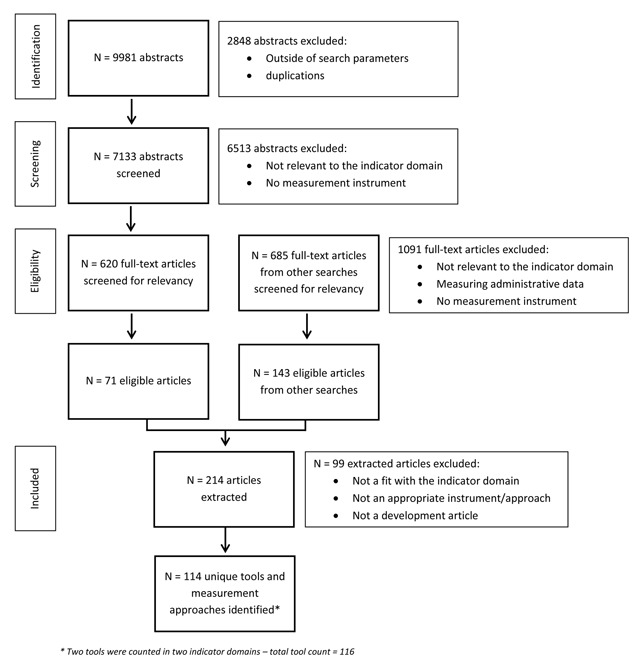
Prisma flow chart.

Figure 1 shows details of the number of abstracts screened, considered relevant/excluded, and included for full review.

## Results

### Delphi Results

Seventeen individuals participated in three rounds of Delphi surveys to identify priority indicator domains for measurement. In the first round, consensus was reached on 15 indicator domains (i.e., ≥ 75% of participants ranked them as either 1 or 2 for relevance and appropriateness). Participants suggested 36 additional domains. These were themed and merged where appropriate to produce 38 indicator domains for round 2. In round 2, the panel reached consensus on 29 indicator domains; the panel agreed that 16 of these indicator domains were relevant/appropriate; 13 were irrelevant/inappropriate; and no consensus was reached on nine indicator domains. These nine indicator domains were submitted to a third round. After three rounds, the panel reached consensus on 37 indicator domains; 16 were considered relevant and used for the systematic literature search for measurement instruments. Twenty-one indicator domains were considered irrelevant and removed. No consensus was reached for indicator domains for Principle 9 focusing on governance.

### Systematic Review Results

The systematic review for the 16 indicator domains yielded a total of 7,133 abstracts. From those, we retrieved 114 unique tools that we considered relevant for measuring the state of integrated care.

Table 1 shows the review results for each of the 16 domains. Some domains were reviewed together given their common characteristics and search terms. Two tools were applicable for two domains. We added a domain “Overall Integration” to capture tools that reflected three or more domains.

**Table 1:** Number of abstracts screened and tools identified by domain.

Domain		Total # abstracts screened	Total # full-text articles	Total # of tools^2^

Principle 1	Coordinated transitions in care across the continuum of care^1^ (transferring care from one area to another)	298	195	17
	Client care is coordinated between sectors and providers within the health system and with supporting services such as education and social services	610	97	14
Principle 2	Patient and/or family involvement in care planning for all patients	569	128	34
Principle 3	Primary care network structures in place (e.g., family health teams, primary care networks, GP Divisions, inner city PHCs)	118	23	8
Principle 4	Team effectiveness	198	83	12
	Use of shared clinical pathways across the continuum of health care (e.g., diabetes, asthma care) and geography^1^	957	229	7
	Individualization of care pathways for patients with co-morbidities			
Principle 5	Performance measurement domains and tools in place^1^	1657	99	2
	Clinical outcomes being measured			
	Data tracked and shared	410	47	0
Principle 6	Data (e.g., administrative, performance, clinical) tracked and shared with stakeholders^1^	315	107	1
	Shared patient electronic charts across continuum of care accessible to patients			
	Data collected is used for service planning	554	68	1
Principle 7	Organizational goals and objectives aligned across sectors	483	50	1
Principle 8	Physician integration within care teams and across sectors	560	53	6
Principle 10	Attainment of goals and objectives are supported by funding and human resource allocation	404	39	1
Overall integration; tools that measure several constructs of integration		0	87	12

**Total**		**7133**	**1305**	**116**

^1^ Overlap in domains; screened together. ^2^ Total number is higher as two tools were appropriate for two domains.

#### Summary of tools

Appendix 1 provides details on the instruments for each indicator domain including concepts measured, setting and sample tested, and psychometric properties where available. The majority of the instruments (94) were questionnaires; other types of instruments included checklists, toolkits, observational tools, and indicators. Ninety-two of the instruments were based on self-report, the other 22 were completed by either an external party or the data was collected from multiple sources (e.g., both the patient and provider). Fifty-six of the instruments were completed by providers, 42 by patients, 10 by administrators, and six by either administrators and providers or patients and providers.

All but eight of the tools came from the peer-reviewed literature, and 110 of the instruments were developed in a healthcare setting. The other four instruments were developed for virtual teams, in the community, or they did not specify. A large number of tools were developed and tested with a specific population (e.g., mental health, pediatrics) but could potentially be adapted for use in the general population. Some of the tools have extensive psychometric properties while others require further testing. The table also lists the number of citations for each instrument to give a sense of a tool’s use.

In the following, we provide more details on tools found under each domain.

### Principle 1: Comprehensive Services across the Care Continuum

#### Coordinated transitions across the continuum of care

Coordinated care transitions was one of two indicator domains identified under principle one, *comprehensive services across the care continuum*. This domain aims to capture the adequacy and continuity of transitional care within and between acute care, primary care, and different community care services and settings. We found 17 instruments that measure continuity of transitional care across the care continuum. Most tools (n = 14) were developed for community/primary care settings [28,29,30,31,32,33,34,35,36,37,38,39,40,41], one was developed in acute care [42], and two in both, primary and acute care [43,44]. The Care Transitions Measure (CTM, [43]) has a number of modifications [45,46].

Many of the tools focus on processes and measure a range of aspects such as timeliness of information transfer, provider continuity, provider-patient interaction and transition planning or the quality of care transition more generally as experienced by the patient. A few tools measure structural components such as transition policies or existence of care plans.

#### Client care is coordinated between sectors and providers within the health system and with supporting services such as education and social services

The second indicator domain under principle one measures the coordination of client services across different sectors, e.g., health and social services coordination. The search yielded 14 instruments that measure intersectoral coordination along a continuum from loose linkages to close collaboration. Most instruments are questionnaires and were created or tested in a health care setting or with health-related outcomes. Intersectoral coordination is captured by variables such as: connections between partnering organizations [47,48,49,50]; social networks [51,52]; interagency linkages [53,54,55,56]; depth of integration [57,58]; and level of system integration and change [59]. Morrissey et al. 1994 [51] developed two instruments appropriate for this domain. Collectively these tools offer a meaningful way to assess the quality and strength of connections between service areas that cross the health and social care sector.

### Principle 2: Patient Focus

#### Patient and/or family involvement in care planning for all patients

This indicator domain focuses on the patient and/or family at the center of care and having them involved in decision-making. This was the only domain under principle two, *patient focus*; however, it covered a broad topic area. Out of all 16 indicator domains, patient and family involvement resulted in the largest number of instruments.

We found 34 instruments that were all created or tested in a health care setting. The majority, 25 of the instruments, are completed by patients and/or families [31,60,61,62,63,64,65,66,67,68,69,70,71,72,73,74,75,76,77,78,79,80,81,82,83,84], the rest are completed by physicians or other health care professionals [85,86,87,88,89], or by both, patients and physicians [90,91,92]. The 30-item Kim Alliance Scale (KAS) [72] was revised (KAS-R) to create a shorter 16-item questionnaire with the same scales [93].

The instruments measure a range of structure, process and outcomes areas mainly from the patient/family perspective such as: 1) patient experiences with care such as administrative processes or customer service aspects; 2) patient satisfaction with various aspects of care such as doctor-patient consultation; 3) quality of care often in relation to patient education and respect received; 4) family involvement in care as expressed, for example, by information received; 5) shared decision-making/involvement with decision-making as a way to participate in the care process; 6) satisfaction with decision made; 7) communication including things such as communication style and preferences; and 8) level of empowerment and empathy. Most instruments contain items in several of these areas allowing for a comprehensive assessment of the patient and family perspective.

### Principle 3: Geographic Coverage and Rostering

#### Primary care network structures in place

Primary care network structures in place was the only indicator domain identified under principle three, *geographic coverage and rostering*. This domain recognizes that health systems integration cannot be achieved without well-developed primary care structures (such as integrated service delivery networks). We found eight questionnaires that measure general structural components [94,95,96,97] or specific areas of primary care, such as the medical home [98,99,100], palliative care [101], and child services [102]. The Medical Home Index (MHI [100]) also has a short version [103,104].

We highlight the Instrumento de Avaliação da Coordenação das RAS pela APS (COPAS) [97] because it is one of the only two unique instruments we found through the search of the Brazilian database. Originally developed in Portuguese, the COPAS is a 78-item questionnaire to assess the coordination of integrated health service delivery networks in primary health care [97]. The COPAS has five dimensions: 1) population, 2) primary health care, 3) support systems, 4) logistic systems, and 5) management systems. The instrument has also been translated into English, the Tool for Assessment of the Coordination of Integrated Health Service Delivery Networks by the Primary Health Care, and has been validated in a primary health care context [105].

### Principle 4: Standardized Care Delivery through Interprofessional Teams

#### Team effectiveness

Team effectiveness was one of three indicator domains under principle four, *standardized care delivery through interprofessional teams*. Team effectiveness, including team performance, represents the effectiveness of interprofessional teams involved in integrated health systems. High performing teams have been a prominent topic for organizational development for many years, and there is no shortage of instruments to measure various aspects of team performance. Recognizing that one type of health provider is rarely able to manage all aspects of complex patients, team-based care has gained much traction in health care [12].

From the broader team literature, we identified 12 instruments we considered relevant to the integration context. Nine instruments were from the health care sector [106,107,108,109,110,111,112,113,114]; the two virtual team questionnaires were from sectors such as technology and agriculture [115,116]; one instrument came from the grey literature [117]. Most of the 12 instruments were questionnaires; one instrument used observational methods to measure teamwork in a surgical setting [113]. Five instruments were part of larger and more in-depth questionnaires [106,108,110,111,115].

These instruments were specifically designed to assess interprofessional teams in health care or teams working in a virtual context. We included instruments that measure the effectiveness of virtual teams as this seemed relevant to integrated care where services are often dispersed. The instruments measure team effectiveness as team perception of their performance, overall team productivity, efficiency and ability of team members to complete their work assignments. Some instruments measure factors that contribute to team effectiveness such as team cohesion, individual well-being and use of resources; or both.

#### Use of shared clinical pathways across the continuum of health care and geography; and Individualization of care pathways for patients with co-morbidities

These two indicator domains were analyzed together as they are similar concepts that could not be distinguished in the screening stage. They focus on if and how shared clinical pathways are used across the continuum of healthcare and geography and on the individualization of care pathways for patients with co-morbidities.

We found five relevant instruments for the shared clinical pathways domain [118,119,120,121,122] and three for the domain that measures individualization of care pathways [123,124,125]; none of them specifically included geography as a component. Four instruments are completed by healthcare management or physicians [118,119,121,122] while two evaluate clinical pathways from the patient perspective [120,123].

These instruments can assist with creating integrated care pathways [125] and evaluating the quality of care pathways and their impact on patient experience [119,124,126]. Shared care pathways are an effective mechanism to create consistency and continuity of care across team members [127]. These instruments will likely gain importance where care pathways cross organizations and care sectors.

### Principle 5: Performance Management

#### Performance measurement indicators and tools in place and Clinical outcomes being measured

These two indicator domains could not be separated in the literature and were therefore analyzed together. Both aim to capture if relevant structures and processes are in place for ongoing quality monitoring. Some authors have identified key aspects for successful performance measurement systems including clear definitions and parameters for indicators, and appropriate feedback loops and mechanisms for reporting [128,129,130]. However, we found only two actual instruments: The Medical Home Index (MHI) [100] includes a number of themes that speak to data management and quality improvement structures. The second instrument, the Índice de Responsividade do Serviço (IRS) (Health Services Responsiveness Index – SRI) is available in Portuguese [131]. The 160-item questionnaire measures Health System Responsiveness to user’s expectations in two areas 1) patient orientation including components that influence patient satisfaction but are not directly connected with health care (agility, social support, facilities and choice) and 2) personal respect including dignity, confidentiality, and autonomy.

#### Data tracked and shared with stakeholders

The third indicator domain under principle five, *performance management*, aims to measure if data is being tracked and shared with stakeholders (e.g., clinicians, staff, policy makers, decision-makers) within health systems. We found no instruments that specifically measures this domain.

### Principle 6: Information Systems

#### Shared information systems across sectors, Shared patient electronic charts across continuum of care accessible to patients and Data collected is used for service planning

These were the three indicator domains identified under principle six, *information systems*. The first two indicator domains capture if information systems exist that are shared across the health system as well as with other sectors such as social services and justice. Furthermore, if such systems are accessible to patients. The only instrument we found for these domains was by Chou et al. 2010 [132]. It consists of structured and open-ended survey questions to evaluate an “internet-based wellness portal” in primary care. The portal provided patients electronic access to their personal health records and resources such as educational content, secure messaging, appointment management, and prescription refills.

The third indicator domain under the *information systems* principle measures if the data collected is used for service planning. The search yielded one instrument, a semi-structured telephone interview guide [133]. Questions focus on types of data used most often, for what purpose and to what capacity, and why data is not being used [133].

### Principle 7: Organizational Culture and Leadership

#### Organizational goals and objectives aligned across sectors

The only indicator domain identified under the principle of *organizational culture and leadership* aimed to assess if there is alignment of organizational goals and objectives across not only the health care system but across sectors such as social services and education. We found one instrument for this indicator domain. The Organizational Culture Assessment Instrument (OCAI) is based on the Competing Values Framework, the dominant theoretical model for assessing organizational culture [134]. The OCAI consists of six items (dominant characteristics, organizational leadership, management of employees, organizational glue, strategic emphasis, and criteria of success), each with four alternatives that reflect four culture types (hierarchy culture, market culture, clan culture, and adhocracy culture). For each of the six items, 100 points are divided between the four culture alternatives; the scores are used to create an organizational culture profile and determine cultural alignment including leadership styles across sectors.

### Principle 8: Physician Integration

#### Physician integration within care teams and across sectors

Physician integration into the broader system, a prominent topic in the late nineties [17], continues to be an important integration issue [135]. For the *physician integration* indicator domain, the only indicator domain for principle eight, we specifically focused on instruments that measure integration between physicians and the health system and integration of physicians within a health care team. Instruments measuring collaboration among team members more generally were included in the *team effectiveness* indicator domain. Instruments measuring integration with patient and families were included in the *patient focus* indicator domain.

We found six relevant instruments [111 (2 instruments), 136,137,138,139]. Newer instruments primarily measure physician integration in the context of provider collaboration (e.g., pharmacists, nurses) rather than physician integration into the broader health system. Given the strong role of physicians in primary care, it makes sense to strengthen and evaluate collaboration between physicians and other health providers for the continuous improvement of quality, safety, and the patient-provider experience [135]. Physician integration is essential to improving care delivery and service planning in the rapidly changing healthcare landscape [17,135]. Some authors have highlighted the integrative function of primary care, arguing that primary care should be “…the starting point from where to improve and integrate care” [12, p.3].

### Principle 10: Financial Management

#### Attainment of goals and objectives are supported by funding and human resource allocation

A single indicator domain was identified for the *financial management* principle. This indicator aims to capture if there is alignment between organizational goals and objectives and how resources are being used. The search yielded one instrument.

The questionnaire by Bradford et al. 2000 [140] measures how resources are allocated and how effective the allocation processes are. Resource allocation best practice questions include priority-setting methods such as needs-assessments, grants making such as targeted requests for funding, service monitoring, and outcome assessment.

### Overall Integration Instruments

The final indicator domain, overall integration, includes instruments that measure health systems integration more generally or that measure three or more of the 16 indicator domains identified.

We found 12 instruments; ten questionnaires target patients, practitioners, managers/leaders, and staff [14,141,142,143,144,145,146,147,148,149]. Two instruments [1,150] use a set of indicators to measure the degree of implementation of integration components. These tools capture some of our 16 indicator domains for which we were unable to find specific instruments. For example, the questionnaire by Gillies et al. 1993 [145] is well established and validated and measures perceived system integration across a number of dimensions such as, alignment of support services, organizational culture, strategic planning, quality assurance, information systems, financial management and resource allocation, and physician integration. This instrument was further developed into the integration scorecard by the same team [143].

Similarly, the Clinical Microsystem Assessment Tool [146] has 10 scales that align with many of the 10 key principles of health systems integration [17] such as culture, organizational support, patient focus, staff focus, interdependence of the care team, information and information technology, process improvement, and performance patterns. The Whole System Measures (WSM) is noteworthy because it not only includes 13 indicators across multiple domains but also recommends measurement methods for each of the 13 indicators. It was developed by the Institute of Healthcare Improvement to promote the use of a “balanced set of system-level measures… to evaluate health systems overall performance” [150, p. 1].

## Discussion

The aim of this knowledge synthesis was to identify meaningful and relevant integration indicator domains and to search for and select appropriate instruments to measure these domains. Building on our previous work [17], we used our ten key integration principles as the framework to prioritize measurement areas and select relevant tools.

Given the nature of the concepts under study, a substantial number of potential indicator domains could be generated for consideration. Delphi is a recognized technique to build consensus amongst experts [26]. In this study, panel members iteratively reviewed indicator domains. Through this process, we were successful in reaching consensus on 16 indicator domains considered highly relevant for measuring progress towards integrated care. The indicator domains span nine of the ten key principles for integration [17], confirming the enduring relevance of these principles. The group reached no agreement on indicator domains for governance. Conceptually, there was no doubt that governance was important, but participants found it difficult to identify measurable indicator domains. Overall, the Delphi process was useful as it helped to identify priority areas for inclusion of instruments in our systematic review.

The subsequent literature review revealed 114 unique instruments that measure various aspects of the 16 domains. The vast majority of instruments found were self-report questionnaires that were completed either by the health care provider or the patient. A popular choice due to the ease of implementation, the limitations of self-report tools have been well recognized. Issues include response bias, recency effects or time pressures. Such reports also greatly depend on a subject’s ability to be insightful, accurate, and honest in their assessment [151]. A broader range of instruments is required to offer assessments of integrated care based on various data.

Over 50% of instruments found measure care coordination across the continuum/sectors, and patient and family involvement. The findings are consistent with other reviews that have uncovered a vast number of instruments related to the concepts of care coordination and patient-centred care [10,20]. This is perhaps not surprising; these domains are the focus for many health care system reforms as progress in these areas directly influence patient care and experience [152].

We only found 14 instruments for the nine indicator domains that related to primary care network structures, performance monitoring, shared information systems, data used for service planning, and organizational alignment. Others have described these domains as functional, system, organizational or normative dimensions of integrated care and have attested to their significance for successful integration [12,22]. The lack of measurement tools in these domains is consistent with findings from the literature. For example, in Bautista’s comprehensive review [10], less than 8% of tools touched on these dimensions. Similarly, the findings from Lyngsø et al. 2014 [2] would suggest that these remain poorly measured aspects of integrated care, pointing to an important evidence gap.

Lastly, we uncovered 12 instruments that measure multiple indicator domains. Some of these instruments simply measure three or more domains while others aim to capture overall integration of a system. Lyngsø et al. [2] have reflected on the benefits of instruments that measure several interdependent dimensions of a complex concept. These “overall integration” instruments are particularly useful for a broad assessment of the overall state of a system. In contrast, domain specific instruments allow measurement of targeted areas that may be the focus of integration strategies.

We assessed the quality of the articles included in the review but did not attempt to systematically evaluate the quality of the instruments. Based on a recent review [10] and our cursory analysis, we expect the quality to range widely. Specifically, many of the instruments were not tested for psychometric properties to support instrument validation. Future research should focus on developing, testing, and validating measures for all domains of health system integration. Similar observations have been made in other evolving fields that lack measures with robust psychometrics [153,154]. Poor reporting may contribute to the quality gap, prompting some researchers to demand clear guidelines for reporting of survey research [155]. There is no question that high quality instruments are essential and should be the preferred choice for measurement purposes. However, instruments validated in one context may not necessarily be valid when applied to a different context [2]. It was interesting to note that the most frequently used instruments (as reflected by number of google citations) were not necessarily of higher quality. This may indicate that the choice of instruments is often influenced by other considerations such as fit with context and the strategy to be evaluated or length and ease of instrument completion.

This project was a partnership between Canada and Brazil. We hoped that expanding the scope beyond Canada would make the work more relevant and universally more applicable. The search of Portuguese databases yielded two unique instruments that were a nice addition to this inventory. This instrument compilation contributes to the growing research concerned with measuring progress towards integrated care (e.g., 2,3,10,18,19,20,22). Collectively, these studies have uncovered several hundred instruments that measure various components of integrated care. While these inventories offer easy access to available instruments, they pose the formidable challenge of how to select the most appropriate instrument from this vast collection. Continued progress towards integrated care will depend much on our ability to contrast and compare the success of strategies across different levels and context. This can only be achieved through a consolidated measurement approach. We support the call for a unified measurement framework, including recommendations on indicators and measurement instruments [13]. Being able to evaluate the success of integration strategies in a consistent way will ultimately lead to better health system design and improved health outcomes for patients.

### Strengths and limitations of this study

Our review has a number of strengths and limitations. We used our previously established framework of the 10 key principles in combination with a consensus approach to select the measurement domains considered most relevant by integration experts. This helped guide the search and selection of instruments. In contrast to other reviews, we included a grey literature search. While the yield was not substantive, instruments published in the grey literature were easy to use as they tended to include user manuals. Easy accessible, user-friendly resources are essential to promote measurement. On the other hand, grey literature reports can easily be missed as they are not always well indexed or posted on easily accessible websites, leading to important omissions. As typical for these kinds of knowledge syntheses, finding the right search terms was challenging and required an iterative approach of searching and refining. Despite ongoing refinement of the search strategies, the literature searches resulted in a vast quantity of literature to examine. Also, the search only included literature up to 2014 so we may have missed some recent instruments. We had numerous people working on different indicator domains providing the opportunity for deviations in our processes. To mitigate these risks, we put in place checks and balances including audits, tracking of decision-making, frequent discussions, and a review of the final report.

## Conclusion

This study has identified over 100 unique instruments that measure 16 different indicator domains considered relevant for integrated care. The majority of instruments were self-report questionnaires that measure care coordination, patient and family involvement, and team effectiveness. In contrast, there were few tools in the domains of performance measurement and information systems, alignment of organizational goals and resource allocation. This remains an area for future research as these domains are relevant for the success of integrated care [10,17,22]. The search yielded 12 tools that measure overall integration or three or more indicator domains. In the absence of more targeted measures for some domains, these overall integration instruments fill an important gap.

Overall, there is a need to develop instruments other than questionnaires to use a broader range of data for measuring integration and integration outcomes. Indicators derived from administrative databases may fill an important gap here and have been the focus of some recent studies [18,19]. Existing instruments would benefit from further psychometric testing and validation in a range of contexts to enhance applicability of the tools.

## Additional File

The additional file for this article can be found as follows:

10.5334/ijic.3931.s1 Appendix Details of Instruments.
