# Supplementary material for: Indicators and Measurement Tools for Health Systems Integration: A Knowledge Synthesis
Source: Int J Integr Care. 2017 Nov 13;17(6):4. doi: 10.5334/ijic.3931 (PMC5854167; doi:10.5334/ijic.3931)
Supplement: Appendix — Details of Instruments. [file ijic-17-6-3931-s1.pdf]

Appendix 1: Details of Instruments

Principle 1: Comprehensive Services across the Care Continuum 34

Coordinated transitions in care across the continuum of care 34

Client care is coordinated between sectors and providers within the health system and with supporting services such as education and social services 43

Principle 2: Patient Focus 49

Patient/family involvement in care planning for all patients 49

Principle 3: Geographic Coverage and Rostering 66

Primary care network structures in place 66

Principle 4: Standardized Care Delivery through Interprofessional Teams 70

Team effectiveness 70

Use of shared clinical pathways across the continuum of health care and geography; and Individualization of care pathways for patients with co-morbidities 77

Principle 5: Performance Management 80

Performance measurement indicators and tools in place and Clinical outcomes being measured 80

Data tracked and shared with stakeholders 81

Principle 6: Information Systems 81

Shared information systems across sectors 81

Shared patient electronic charts across continuum of care assessable to patients 81

Data collected is used for service planning 82

Principle 7: Organizational Culture and Leadership 82

Organizational goals and objectives aligned across sectors 82

Principle 8: Physician Integration 83

Physician integration within care teams and across sectors 83

Principle 10: Financial Management 85

Attainment of goals and objectives are supported by funding and human resource allocation 85

Overall Integration Instruments 86

Principle 1: Comprehensive Services across the Care Continuum

Coordinated transitions in care across the continuum of care

Author	Name	Description	Setting & Sample	Psychometrics
Bonomi et al. 2002<br>Google Scholar citations: 313<br>Quality rating: 6/15	Assessment of Chronic Illness Care (ACIC)	28-item questionnaire to measure quality improvement in chronic illness care:<br>• 6 subscales: 1) community linkages, 2) self-management support, 3) decision support, 4) delivery system design, 5) information systems, and 6) organization of care	USA<br>n = 108 organizational teams from health systems	Paired t-tests measured changes in subscale scores<br>Pearson correlation coefficients used to evaluate the follow-up ACIC scores
Coleman et al. 2002<br>Google Scholar citations: 161<br>Quality rating: 15/15	Care Transitions Measure (CTM)	15-item questionnaire to measure the quality of care transitions across healthcare settings:<br>• 4 subscales: 1) information transfer, 2) patient and caregiver preparation, 3) support for self-management, and 4) empowerment to assert preferences<br>• Patient-centered rather than provider centered	USA<br>n = 49 patients and care givers in 6 focus groups of 7-10	Face-validity assessed in pilot-testing<br>Construct validity
Coleman et al. 2005 (2nd development article)<br>Google Scholar citations: 221<br>Quality rating: 14/15	Care Transitions Measure (CTM)	Revised 15-item questionnaire to measure the quality of care transitions across healthcare settings:<br>• 4 revised subscales: 1) critical understanding, 2) preferences important, 3) management preparation, and 4) care plan	USA<br>n = 200 patients discharged from urban hospitals	Construct and discriminant validity<br>Factor analysis: 4 factors<br>Cronbach’s α = 0.93 (overall scale)

Author	Name	Description	Setting & Sample	Psychometrics
Parry et al. 2008<br>Google Scholar citations: 91<br>Quality rating: 12/15	Care Transitions Measure -3 (CTM-3)	Revised 3-item questionnaire to measure the quality of care transitions across healthcare settings:<br>• The 3-item CTM can predict the 15-item CTM score<br>• The goal of the CTM-3 was to reduce to response burden of the CTM	USA<br>n = 225 African American, Hispanic American, or rural adults living in areas with populations less than 10,000 hospitalized in the last 12 months	Predictive validity for 3-item scale<br>Factor analysis: 4 factors confirmed for 15-item scale<br>Cronbach's α's for 15 item scale = 0.94 (African Americans); α = 0.93 (Hispanic Americans); α = 0.96 (rural dwelling subjects); α = 0.93 to 0.95 (range of demographics)
Durbin et al. 2004<br>Google Scholar citations: 59<br>Quality rating: 13/15	Alberta Continuity of Services Scale-Mental Health (ACSS-MH)	30-item questionnaire to measure perceived consumer continuity of care across settings and providers:<br>• 3 subscales 1) system access, 2) interpersonal aspects of care, and 3) team functioning and outreach	Canada<br>n = 215 consumers of community and outpatient mental health services	Factor analysis: 3 factors<br>Cronbach's α = 0.74 to 0.88 (range of subscales)<br>Bivariate correlations between subscale scores = 0.46 to 0.58
Farmanova, Grenier & Chomienne 2013<br>Google Scholar citations: 0<br>Quality rating: 7/15	Mental Health Services in Family Health Teams (MHS-FHT) tool	37-item provider questionnaire and 35-item patient questionnaire to measure the provision of mental health services in family health teams:<br>• Questionnaires measure 6 subscales: 1) acceptability, 2) accessibility, 3) appropriateness, 4) continuity, 5) effectiveness, and 6) comprehensiveness of care	Canada<br>n = 22 providers and patients from family health integration networks	Pilot tested using cognitive debriefing

Author	Name	Description	Setting & Sample	Psychometrics
Graetz et al. 2014<br>Google Scholar citations: 16<br>Article quality rating: 7.5/15	Questionnaire (no name specified)	8-item questionnaire to measure care coordination. Participants were asked questions about 2 care transition situations:<br>• Questions measure: care transitions between clinicians, care transitions across delivery sites, fill availability of medical information, timely information transfer, agreement of treatment plan, and agreement of roles and responsibilities	USA<br>n = 1,869 adult primary care clinicians (general practitioners, nurse practitioners, physician assistants) in an integrated delivery system hospital	Not reported
Grimmer & Moss 2001<br>Google Scholar citations: 54<br>Quality rating: 11/15	Prescriptions, Ready to re-enter community, Education, Placement, Assurance of safety, Realistic expectations, Empowerment, Directed to appropriate services (PREPARED)	Tool to measure community consumers satisfaction with discharge planning activities:<br>• 4 key process subscales: 1) information exchange, 2) medication management, 3) preparation for coping after discharge, and 4) control of discharge circumstances	South Australia<br>n = 8 medical and surgical ward charge nurses<br>n = 26 medical and surgical ward staff	Content, divergent, construct, and concurrent validity

Author	Name	Description	Setting & Sample	Psychometrics
King, Rosenbaum & King 1996<br><br>Google Scholar citations: 185<br><br>Article quality rating: 14/15	Measure of Processes of Care (MPOC)	56-item questionnaire to measure parents' experiences of being included and supported in their children's care:<br>• Looks at the extent that health care professionals exhibit specific behaviours<br>• 5 subscales: 1) enabling and partnership, 2) providing general information, 3) providing specific information about the child, 4) co-ordinated and comprehensive care for child and family, and 5) respectful and supportive care	Canada<br><br>Pilot-test<br>n = 653 parents of children with disabilities recruited from participating ambulatory clinics	Discriminant validity<br><br>Factor analysis: 5 factors<br><br>Cronbach's α = 0.81 to 0.96 (range of subscales)<br><br>Test-retest reliability with intraclass correlations coefficients = 0.78 to 0.88 (range of subscales)
King, King & Rosenbaum 2004<br><br>Google Scholar citations: 141<br><br>Article quality rating: 14/15	Measure of Processes of Care refined (MPOC-20)	Revised 20-item questionnaire to measure parents' experiences of being included and supported in their children's care:<br>• The MPOC-56 was reduced to a 20-item questionnaire and the labelling of the response scales was improved<br>• The 5 subscales from the MPOC-56 are the same in the MPOS-20	Canada<br><br>Re-examined data from previous studies:<br>Pilot study: n = 653 parents of children with disabilities<br>Field test: n = 151 parents<br>Reliability study: n = 29 parents<br>Validity study: n = 14 parents<br>Cross-Sectional study: n = 164 parents<br><br>New data<br>n = 494 parents of children with disabilities	Concurrent validity between MPOC and MPOC-20<br><br>Discriminant validity<br><br>Factor analysis: 5 factors<br><br>Cronbach's α =0.83 to 0.90 (range of subscales)<br><br>Intraclass correlation coefficients between the scales = 0.56 to 0.87

Author	Name	Description	Setting & Sample	Psychometrics
Granat et al. 2002<br>Google Scholar citations: 48<br>Quality rating: 11/15	Measure of the Processes of Care (MPOC-28)	Revised 28-item questionnaire to measure parents' experiences of being included and supported in their children's care:<br>• The MPOC-28 (based off the MPOC-20) was developed as a national evaluation instrument for Sweden's children disability services<br>• To be appropriate for the Swedish context, 5 additional questions were kept from the MPOC-56 and 3 new questions were added	Sweden<br>n = 2,458 families with children under 18 who had used habitation services for at least 1 year	Factor analysis: 5 factors<br>Cronbach's α = 0.85 to 0.91 (range of subscales)
Le Bas, King & Block 1998<br>Google Scholar citations: 6<br>Quality rating: 9.5/15	Questionnaire (no name specified)	11-item questionnaire to measure the change in mental health staff's opinions before and after transitioning to a complex integrated service:<br>• The questionnaire measures: perceptions of staff, cohesion, communication, continuity of care, debriefing, enthusiasm, focus, line accountability, economy of meetings, staff development, simplicity of structure, work allocation, and sharing	Australia<br>n = 30 medical, nursing and allied health staff of adult mental health services<br>Psychometric properties of the scale tested with 2 datasets:<br>n = 223 cases from 5 successive administrations at the Peninsula Health Care Network Psychiatric Services<br>n = 71 cases from a single administration at a mental health services in Brisbane	Factor analysis: unidimensional<br>Cronbach's α = 0.88 (n=71) to 0.90 (n=223)

Author	Name	Description	Setting & Sample	Psychometrics
Lemmon & Shuff 2001<br>Google Scholar citations: 1<br>Quality rating: 15/15	Mental Health Professional HIV/AIDS Point Prevalence & Treatment Experiences Survey Part II	34-item questionnaire to measure mental health system integration for patients with HIV:<br>• 4 categories of questions: 1) mental health system (MHS) integration with primary care physicians, 2) MHS integration with care coordination sites, 3) MHS integration with other mental health centres, and 4) internal integration of HIV care into the MHS itself	USA<br>n = 51 mental health staff who had participated in the Indiana integration of care project and had seen HIV/AIDS patients in the past year	Face validity<br>Cronbach's α = 0.57 to 0.90 (range of subscales)
Martz & Gerding 2011<br>Google Scholar citations: 4<br>Quality rating: 10.5/15	Questionnaire (no name specified)	38-item questionnaire to measure similarities and differences in perceptions of nursing care between nursing homes (NH) and skilled nursing facilities (SKF):<br>• The questionnaire measures: collaboration, overall experience of NH/SKF practices, benefits and barriers to care	USA<br>n = 200 nursing home and hospice care staff	Face and content validity<br>Cronbach's α = 0.86 (overall scale); 0.82 to 0.93 (range of subscales)
McGuiness & Sibthorpe 2003<br>Google Scholar citations: 31<br>Quality rating: 11/15	Client Perceptions of Coordination Questionnaire	32-item questionnaire to measure coordination of health care perceived by the client:<br>• 6 subscales: 1) acceptability, 2) received care, 3) GP, 4) nominated provider, 5) client comprehension, and 6) client capacity.	Australia<br>n = 1,193 adults with complex and chronic health care needs who were participants in a RTC of coordinated care	Face and content validity<br>Construct validity "known groups" approach<br>Factor analysis: 6 factors<br>Cronbach's α = 0.92 (overall scale); 0.31 to 0.86 (range of subscales)<br>Spearman correlation coefficient = 0.30 to 0.70 (range for pairs of subscales)

Author	Name	Description	Setting & Sample	Psychometrics
Safran et al. 2006<br>Google Scholar citations: 153<br>Quality rating: 11/15	The Massachusetts Ambulatory Care Experiences Survey (ACES)F	11-item questionnaire to measure patients' experiences with medical groups and individual physicians:<br>• 2 subscales: 1) quality of physician-patient interactions and 2) organizational features of care	USA<br>n = 9,625 adult medical groups on commercial health plans and Medicaid	Physician-level reliability = 0.70 to 0.90 (Spearman Brown Prophecy Formula)
Sawicki et al. 2009<br>Google Scholar citations: 157<br>Quality rating: 15/15	Transitions Readiness Assessment Questionnaire (TRAQ)	29-item questionnaire to measure the transition readiness from pediatric to adult healthcare for youth with special health care needs:<br>• 2 subscales: 1) skills for self-management and 2) skills for self-advocacy	USA<br>n = 192 youth with special health care needs transitioning from pediatric to adult health care aged 16-26	Factor analysis: 2 factors<br>Cronbach's α = 0.92 and 0.82 (subscales)
Schaefer, Cronkite & Ingudomnukul 2004<br>Google Scholar citations: 8<br>Quality rating: 12/15	Continuity of Care Practices Survey (CCPS-P)<br>Continuity of Care Practices Survey (CCPS-I)	Two parallel 23-item questionnaires to measure self-report assessment of continuity of care practices at the program and individual level:<br>• 4 subscales: 1) provider continuity, 2) maintain contact, 3) connect to resources, and 4) coordinate care	USA<br>n = 129 directors and coordinators of intensive inpatient/residential outpatient programs (CCPS-P)<br>n = 835 counsellors of patients in 28 substance use disorder programs	Discriminant and predictive validity<br>Cronbach's α = 0.61 to 0.85 (range of subscales)<br>Item to sub-scale correlations = 0.19 to 0.41 and 0.51 to 0.70<br>Average inter-item correlation 0.43

Author	Name	Description	Setting & Sample	Psychometrics
Tobon, Reid & Goffin 2014<br><br>Google Scholar citations: 3<br><br>Quality rating: 14.75/15	Continuity of Care in Children's Mental Health (C3MH)	42-item questionnaire to measure continuity of care experienced by families receiving services through the children's mental health system:<br>• 5 scales: 1) collaboration, 2) transitions, 3) provider knowledge, 4) relational interpersonal, and 5) relational consistency<br>• Parent and youth versions were developed	Canada<br><br>n = 364 parents of children and youth recruited from 13 children's mental health agencies<br><br>n = 57 youth piloted the youth version	Convergent and discriminant validity<br><br>Factor analysis: 5 factors<br><br>Cronbach's α = 0.80 to 0.93 (range of subscales)<br><br>Test-retest reliability >0.75
**Grey Literature**				
Center for Health Care Transition Improvement, 2014 (www.GotTransition.org)<br><br>Google Scholar citations: 0<br><br>Quality rating: N/A	Got Transition: Current Assessment of Health Care Transition Activities Tool	8-item self-assessment tool for providers, clinics, or organizations to assess the level of support available for transition from pediatric to adult care:<br>• Assesses 6 core elements for health care transition: 1) transition policy, 2) transition tracking and monitoring, 3) transition readiness, 4) transition planning, 5) transfer of care, and 6) transfer completion<br>• 2 additional questions collect data on youth, young adult, and/or family feedback and leadership<br><br>3 versions were developed: youth to adult, young adults to adult, transition to adult approach (some core elements are slightly different depending on the version)	USA	No psychometrics available<br><br>Six Core Elements and tools were developed based on the American College of Pharmacists , American Academy of Pediatrics, and American Academy of Family Physicians' Clinical Report on Transition, learning collaboratives, and studying innovations in transitions of care in the US and internationally<br><br>Reviewed by over 50 pediatric and adult health care providers, youth, and family members

Author	Name	Description	Setting & Sample	Psychometrics
Center for Health Care Transition Improvement, 2014 (www.GotTransition.org)<br><br>Google Scholar citations: 0<br><br>Quality rating: N/A	Got Transition: Health Care Transition Process Measurement Tool	29-items measure the implementation of the 6 core elements (as listed above) and their dissemination to youth, young adults and their families:<br>• Suggested use is for baseline and follow-up data collection for transition improvement initiatives<br><br>3 versions were developed: youth to adult, young adults to adult, transition to adult approach (some core elements are slightly different depending on the version)	USA	See above

Client care is coordinated between sectors and providers within the health system and with supporting services such as education and social services

Comments	Name	Description	Setting & sample	Psychometrics
Amoroso et al. 2007<br>Google Scholar citations: 4<br>Quality rating: 11.5/15	General Practice Clinical Linkages Interview (GP-CLI)	9-item interview questionnaire to measure the quality of chronic disease related clinical linkages:<br>• Determines the facilitators, impact, and outcomes of practice-based linkages<br>• 3 subscales: 1) shared care/care planning, 2) community access/awareness, and 3) referral/advice	Australia<br>n = 97 general practitioners and practice managers	Concurrent validity<br>Factor analysis: 3 factors<br>Cronbach's α = 0.53 to 0.68 (range of subscales)
Browne et al. 2004<br>Google Scholar citations: 76<br>Quality rating: 11/15	Human Services Network Integration Measure	Instrument to quantitatively measure the scope and depth of integration for each sector and service in a network:<br>• The measurement of the actual or observed versus the expected extent, scope and depth of integration of services within a network<br>• Items depend on the organizations using the measure	Canada<br>n = 2 children's programs (the Healthy Babies, Healthy Children program and the Early Years program)	Content, face, and discriminate validity
Conrad et al. 2003<br>Google Scholar citations: 37<br>Quality rating: 13/15	Partnerships Self-Assessment Survey (PSAS)	Questionnaire to measures progress towards more rational and cost-effective service delivery:<br>• 4 subscales: 1) community health focus, 2) seamless continuum of care, 3) community accountability, and 4) managing under fixed resources<br>• Includes an overall progress score (average scores of the 4 individual progress scores)	USA<br>n = 25 community-based health partnerships	Cronbach's α = 0.91 (overall scale)<br>Correlation coefficients between factors r = 0.60 to 0.80<br>Note: conventional levels of inter-rater reliability was not reached

Comments	Name	Description	Setting & sample	Psychometrics
Fletcher et al. 2009<br>Google Scholar citations: 68<br>Article quality rating: 13/15	National Criminal Justice Treatment Practices Survey (NCJTPS)	2-sector specific questionnaires measure interagency collaboration and integration activities:<br>• Activities were classified as "high" or "low" structure: low = cooperation and coordination; high = collaboration and consolidation<br>• 2 questionnaires: treatment program questionnaire (12- items), corrections program questionnaire (11-items)	USA<br>n = 430 directors, administrators, ad frontline staff from 4 organizational levels of criminal justice and n = 217 addiction organizations	Factor analysis: 2 factors for both surveys
Meredith et al. 2009<br>Google Scholar citations: 18<br>Quality rating: 12/15	Questionnaire (no name specified)	Questionnaire to measure inter-organizational linkages to coordinate services for PTSD:<br>• 2 subscales: 1) the level of mental health integration (how the primary medical care services relate to mental health services) and 2) the extent of community linkages<br>• Intersectoral items were a part of a 2-page survey for medical directors and 4-page for primary care clinicians	USA<br>n = 47 medical directors and n = 154 primary care clinicians (all 201 were members of a Clinical Directors Network)	Not reported

Comments	Name	Description	Setting & sample	Psychometrics
Morrissey et al. 1994<br>Google Scholar citations: 38<br>Quality rating: 11/15	Inter-organizational Network Survey	Questionnaire to measure the change in coordination and centralization of mental health and community agencies:<br>• Measures 3 sets of exchanges between community agencies: 1) client referrals, 2) shared information, and 3) funds exchanges<br>• Exchange relations are measured at two time points<br>• 3 indices measure the structure of relations in each network: 1) density, 2) centralization, and 3) fragmentation	USA<br>n = 6 cities measuring local mental health authorities and community support systems	Not reported
Morrissey et al. 1994<br>Google Scholar citations: 38<br>Quality rating: 11/15	Key Informant Survey	42-item questionnaire to measure how well local agencies are meeting the needs of people with chronic mental illness:<br>• 4 subscales: 1) adequacy, 2) quality, 3) availability, and 4) coordination<br>The questionnaire includes 12 additional items that measure performance.	USA<br>n = 10 cities measuring local mental health authorities and community support systems	Cronbach's α = 0.65 to 0.95 (range of subscales)
Morrissey et al. 1997<br>Google Scholar citations: 92<br>Quality rating: 13/15	ACCESS Evaluation	15-item questionnaire to measure interagency linkages evaluated in a program evaluation:<br>• 2 subscales: 1) perceived accessibility and 2) perceived coordination<br>• Produces a matrix with 6 options: the agency is "sending" or "receiving" information about "referrals", "funds transfers" or "information sharing"	USA<br>n = 875 respondents (usually agency or program director) from ACCESS agencies or programs	Perceived accessibility Cronbach's α = 0.87<br>Perceived coordination Cronbach's α = 0.74

Comments	Name	Description	Setting & sample	Psychometrics
Pagliccia et al. 2010<br>Google Scholar citations: 17<br>Quality rating: 13/15	Questionnaire (no name specified)	3-item questionnaire to rate the importance of 11 health determinants:<br>• 6 subscales: 1) perceived importance of health determinants, 2) intensity of internal engagement, 3) intensity of intersectoral collaboration, 4) network density, 5) betweenness centrality, and 6) betweenness centralization<br>• block modeling techniques (network analysis) to assess how the different sectors group together within the network	Cuba<br>n = 113 policy makers representing different sectors	Not reported
Passalent et al. 2013<br>Google Scholar citations: 0<br>Quality rating: 12/15	The evaluation of the Systems Integration and Change (SIC) quadrant for the balanced scorecard	Integration and change balanced scorecard quadrant to measure the system-level impact of the Advanced Clinician Practitioner in Arthritis Care (ACPAC) program:<br>• System integration was measured by looking at: 1) access to care, 2) integration of extended role practice, and 3) integration with the healthcare system	Canada<br>n = 30 ACPAC program-trained graduates	Content and face validity<br>The questionnaire was pilot-tested on 3 occasions
Reilly et al. 2003<br>Google Scholar citations: 41<br>Quality rating: 10/15	Questionnaire (no name specified)	29-item questionnaire to measure the degree of integration between health and social care provision and general services arrangements:<br>• Measures focus on operational integration, team membership, team process, and team management<br>• Separate sets of indicators were used to identify links with social care and primary health care	United Kingdom<br>n = 317 old age psychiatrists	Not reported

Comments	Name	Description	Setting & sample	Psychometrics
Singer et al. 2012<br>Google Scholar citations: 16<br>Quality rating: 13/15	Patient Perceptions of Integrated Care (PPIC)	20-item questionnaire to measure care coordination and patient centeredness:<br>• 6 subscales: 1) information flow to your doctor, 2) post-visit information flow to the patient, 3) information flow to your specialist, 4) coordination with home and community resources, 5) information flow to other providers in your doctor's office, and 6) patient-centeredness	USA<br>n = 527 patients with 2 or more chronic conditions from 13 primary care clinics	Factor analysis: 6 factors<br>Cronbach's α = 0.62 to 0.80 (range of subscales)<br>Factor correlations = 0.14 to 0.84<br>Intraclass correlation coefficients (patient-level)= 0.03 to 0.00 (not statistically significant)<br>Intraclass correlation coefficients (clinic-level) = 0.57 to 0.09 (not statistically significant)
Tucker et al. 2007<br>Google Scholar citations: 44<br>Quality rating: 9/15	Questionnaire (no name specified)	Questionnaire to measure old age psychiatrists' perspectives of the National Service Framework for Older People (NSFOP) Mental Health Model:<br>• 4 subscales: 1) the range of specialist, 2) mental health provision, 3) the nature of the specialist/generic service interface, and 4) the degree of interdisciplinary/ interagency working)<br>• Indicators for interdisciplinary/ interagency working include: 1) assessment, 2) care planning, and 3) record keeping	United Kingdom<br>n = 318 old age psychiatrists	Not reported

Comments	Name	Description	Setting & sample	Psychometrics
**Grey Literature**				
VicHealth 2003<br>Google Scholar citations: 0<br>Quality rating: N/A	VicHealth Partnership Analysis Tool	35-item checklist to measure agencies' capacity of effective partnership work:<br>• 7 subscales: 1) need for the partnership, 2) choosing partners, 3) making sure partnerships work, 4) planning collaborative action, 5) implementing collaborative action, 6) minimising the barriers to partnerships, and 7) reflecting on and continuing the partnership	Not reported	Not reported

Principle 2: Patient Focus

Patient/family involvement in care planning for all patients

Comments	Name	Description	Setting & sample	Psychometrics
**Patient Centred Care/Experiences with Care**				
Arora et al. 2011<br>Google Scholar citations: 66<br>Article quality rating: 13/15	Assessment of Patient Experiences of Cancer Care (APECC)	33-item questionnaire to measure survivors' perceptions of the quality of follow-up care in the last 12 months:<br>• 30 items make up 10 subscales: 1) getting needed care, 2) timeliness of care, 3) waiting time in physician's office, 4) information exchange, 5) physicians' affective behavior, 6) physicians' knowledge, 7) interaction with nurses, 8) interaction with office staff, 9) health promotion, and 10) coordination of care<br>• The 10 subscales are broken up into 5 categories: 1) access to care, 2) interaction with physicians, 3) interaction with other members of the health care team, 4) discussion of health promotion, and 5) perceptions of coordination of care<br>• The remaining 3 items provide an overall rating of care	USA<br>n = 623 survivors (patients) of leukemia, bladder or colorectal cancer	Factor analysis: 10 factors<br>Cronbach's α = 0.87 (for overall scale); 0.76 to 092 (range of subscales)

Comments	Name	Description	Setting & sample	Psychometrics
Hays et al. 1999<br><br>Google Scholar citations: 252<br><br>Quality rating: 10/15	Consumer Assessment of Health plans Study (CAHPS®) 1.0	28-item questionnaire to measure the quality of care provided by health plans, physician groups, and clinicians:<br>• 4 items measure global ratings: 1) personal doctor, 2) health plan, 3) specialist care, and 4) care received<br>• 24 items measure 10 subscales: 1) getting the care you need, 2) getting care without long wait, 3) communication, 4) enough time spent, 5) prevention, 6) office staff, 7) customer service, 8) reasonable paperwork, 9) finding personal doctor, and 10) referral to specialists<br><br>Multiple versions of the CAHPS have been developed (specific providers, conditions, youth/adult and languages).	USA<br><br>4 samples:<br><br>n = 5,878 adult and child patients on Medicaid from CAHPS demonstration sites<br><br>n = 11,393 adult and child patients with private insurance from CAHPS demonstration sites<br><br>n = 313 adult and child patients on Medicaid from CAHPS field test sites<br><br>n = 539 adult patients with private insurance from CAHPS field test sites	Construct validity<br><br>Cronbach's α for 10 subscales = 0.48 to 0.79 (range for patient on Medicaid); 0.48 to 0.88 (range for patients with private insurance)<br><br>Plan level reliability (ANOVA) for global rating items = 0.13 to 0.77 (range for patients on Medicaid); 0.29 to 0.96 (patients with private insurance)<br><br>Plan-level reliability (ANOVA) for 10 subscales = 0.00 to 0.78 (range for patients on Medicaid); 0.45 to 0.95 (range for patient with private insurance)

Comments	Name	Description	Setting & sample	Psychometrics
Hargraves, Hays & Cleary 2003<br>Google Scholar citations: 202<br>Quality rating: 10/15	Consumer Assessment of Health plans Study (CAHPS®) 2.0 Adult Core Survey	Revised 43-item questionnaire to measure health plans and providers from the consumer's perspective:<br>• 19 are core items routinely reported to consumers<br>• 17 questions are grouped into 5 composites for public reporting: 1) getting care quickly, 2) doctors who communicate well, 3) courteous/helpful office staff, 4) getting needed care, and 5) health plan customer service<br>• 2 global rating items to identify questions that may not apply to all respondents: personal doctor or nurse, specialist, quality of health care, and health plan<br>• Items have been modified from the 1.0 version	USA<br>n = 166,074 privately insured respondents from 306 U.S. health plans	Construct and predictive validity<br>Factor analysis: 5 factors<br>Cronbach's α = 0.51 to 0.86 (range of subscales)<br>Plan-level reliability: 0.88 to 0.95 (5 subscales); 0.82 to 0.96 (global ratings)
Jenkinson, Coulter & Bruster 2002<br>Google Scholar citations: 334<br>Article quality rating: 11/15	Picker Patient Experience Questionnaire (PPE-15)	15-item questionnaire to measure patient experiences of in-patient care.	United Kingdom, Germany, Sweden, Switzerland, and USA<br>n = 62,925 recently discharged acute care patients	Cronbach's α = 0.80 to 0.87 (overall scale for various countries)<br>Item correlations = 0.23 to 0.58
King, Rosenbaum & King 1996<br>Google Scholar citations: 185<br>Article quality rating: 14/15	Measure of Processes of Care (MPOC)	See domain 1.1 for questionnaire details.		

Comments	Name	Description	Setting & sample	Psychometrics
King, King & Rosenbaum 2004<br><br>Google Scholar citations: 141<br><br>Article quality rating: 14/15<br><br>Granat et al. 2002<br><br>Google Scholar citations: 48<br><br>Quality rating: 11/15	Measure of Processes of Care refined (MPOC-20)<br><br>Measure of the Processes of Care (MPOC-28)	See domain 1.1 for questionnaire details.<br><br>See domain 1.1 for questionnaire details.		
Little et al. 2001<br><br>Google Scholar citations: 712<br><br>Article quality rating: 8/15	Questionnaire (no name specified)	21-item questionnaire to measure patient centered doctor consultation:<br>• 5 subscales : 1) communication and partnership, 2) personal relationship, 3) health promotion, 4) positive and clear approach to problem, and 5) interest in effect on life	United Kingdom<br><br>n = 865 patients recruited from 3 general practices	Factor analysis: 5 factors<br><br>Cronbach's α = 0.84 to 0.96 (range of subscales)

Comments	Name	Description	Setting & sample	Psychometrics
Safran et al. 1998<br>Google Scholar citations: 619<br>Quality rating: 12/15	Primary Care Assessment Survey (PCAS)	51-item questionnaire to measure primary care performance:<br>• 11 summary scales are used to measure 7 domains of care: 1) accessibility (organizational, financial), 2) continuity (longitudinal, visit-based),<br>3) comprehensiveness (contextual knowledge of patient, preventive counseling), 4) integration, clinical interaction (clinician-patient communication, 5) thoroughness of physical examinations), 6) interpersonal treatment, and 7) trust	USA<br>n = 7,204 adult employees enrolled in any of 12 health plans offered to commonwealth employees	Content and face validity<br>Five Likert Scaling Assumptions: 1) item-convergent validity, 2) item-discriminate validity, 3) equal item variance, 4) equal item-scale correlations, 5) score reliability
Sixma et al. 1998<br>Google scholar citations: 324<br>Quality rating: 9/15	Quality of Care Through the Patients' Eyes (QUOTE)	40-item questionnaire to measure the quality of care from the patients' perspective:<br>• Questions measure: generic aspects indicators and disease-specific indicators<br>• Quality of care is measured with importance and performance statements<br>Different versions of the QUOTE were developed for different conditions.	The Netherlands<br>n = 287 patients with rheumatic diseases (55 years or older)	Cronbach's alpha = 0.74 to 0.88 (process, structure, and category-specific subscales)
**Patient Satisfaction**				
Meakin & Weinman 2002<br>Google Scholar citations: 119<br>Article quality rating: 11/15	Medical Interview Satisfaction Scale (MISS-21)	21-item questionnaire to measure patient satisfaction with individual doctor-patient consultations:<br>• 4 subscales: 1) distress relief, 2)communication comfort, 3) rapport, and 4) compliance intent	England<br>n = 182 general practice patients	Factor analysis: 4 factors<br>Cronbach's α = 0.92 (overall scale)

Comments	Name	Description	Setting & sample	Psychometrics
		**Quality of Care**		
Damman, Hendriks & Sixma 2009<br>Google Scholar citations: 46<br>Article quality rating: 11/15	Consumer Quality Index Breast Care (CQI-BC)	The CQI-BC measures the specific experiences of patients with breast cancer. The CQI includes 3 parts:<br>• The CQI-BC Experience questionnaire, the CQI-BC Importance Questionnaire, and the Quality Improvement Scores<br>• The main questionnaire is the CQI-BC Experience Questionnaire which includes 152-items in 15-reliable subscales<br>118-items are related to patient experience. The remaining items measure: general items, timeliness of care and results, and the patient's global perspective of the healthcare and healthcare providers.<br>Different versions of the CQI were developed for different conditions.	The Netherlands<br>n = 27 breast cancer patients in 3 focus groups<br>n = 731 breast cancer patients completed the survey	Factor analysis: 15 factors<br>Cronbach's α = 0.68 to 0.93 (range of subscales)
de Kok et al. 2007<br>Google Scholar citations: 27<br>Article quality rating: 8/15	Questionnaire (no name specified)	55-item questionnaire to measure quality of breast cancer care from the patient's perspective:<br>• 6 clusters: 1) education,<br>2) continuity of care, 3) respect for the patient, 4) time schedule, 5) period of admission, and 6) focus on the patient	The Netherlands<br>n = 72 patients with breast cancer	Multidimensional scaling and hierarchical cluster analysis: 6 clusters

Comments	Name	Description	Setting & sample	Psychometrics
de Kok et al. 2010<br>Google Scholar citations: 10<br>Article quality rating: 12/15	Questionnaire (no name specified)	Revised 33-item questionnaire to measure quality of breast cancer care from the patient's perspective::<br>• 5 subscales include: 1) patient education related to postoperative treatment, 2) services by the breast nurse, 3) services by the surgeon, 4) patient education regarding activities at home, and 5) patient education regarding preoperative treatment-related aspects	The Netherlands<br>n = 276 breast cancer patients operated on in last 3-15 months	Factor analysis: 5 factors<br>Cronbach's α = 0.70 to 0.89 (range of subscales)
**Family Involvement in Care**				
Agnew-Davies et al. 1998<br>Google Scholar citations: 149<br>Article quality rating: 9/15	Agnew Relationship Measure (ARM)	26-item questionnaire to measure the client-therapist alliance:<br>• The client and the therapist complete the questionnaire<br>• 3-types of items: 1) the client, 2) the therapist, and 3) the client-therapist relationship<br>• 5 subscales: 1) bond, 2) partnership, 3) confidence, 4) openness, and 5) client initiative	United Kingdom<br>n = 95 clients 5 clinical psychologists involved in psychotherapy sessions in the Second Sheffield Psychotherapy Project for the treatment of depression	Factor analysis: 5 factors<br>Cronbach's α = 0.55 to 0.87 (range of subscales for client scale); = 0.55 to 0.86 (range of subscales for therapist scale)
Ainsworth, Cowan & Trieschman 1998<br>Google Scholar citations: 10<br>Article quality rating: 12/15	Model for family centered group care	59-item questionnaire to test the model fit of a path diagram of family centered group care practice:<br>• 4 subscales: 1) tangible services, 2) maintaining parent/child connections, 3) parental decision making, and 4) staff attitudes	USA<br>Pilot-test<br>n = 239 staff of 18 group care agencies<br>Validation<br>n = 169 staff of 3 New England group care agency programs	Concurrent validity<br>Factor analysis: 4 factors

Comments	Name	Description	Setting & sample	Psychometrics
Galassi, Schanberg & Ware 1992<br>Google Scholar citations: 89<br>Article quality rating: 8/15	Patient Reactions Assessment (PRA)	15-item questionnaire to measure the perceived quality of the patient-provider relationship:<br>• 3 subscales: 1) patient information index (PII), 2) the patient affective index (PAI), and 3) the patient communication (PC) index	USA<br>Study 1 (face validity): n = 17 oncology nurses and counselling students<br>Study 2 (item reduction and scale structure): n = 220 cancer patients receiving treatment<br>Study 3 (confirm factor structure and concurrent validity): n = 197 cancer patients receiving treatment	Concurrent validity<br>Factor analysis: 3 factors<br>Cronbach's α = 0.91 (overall scale); = 0.87 to 0.91 (range of subscales)
Kim, Boren & Solem 2001<br>Google Scholar citations: 42<br>Article quality rating: 12/15	Kim Alliance Scale (KAS)	30-item questionnaire to measure the quality of the therapeutic alliance from the patient's perspective:<br>• 4 subscales: 1) collaboration, 2) communication, 3) integration, and 4) empowerment	USA<br>n = 68 convenience sample of registered nurses who had been patients themselves within the past 2 years	Content, convergent, and divergent validity<br>Factor analysis: 4 factors<br>Cronbach's α = 0.94 (overall scale); 0.71 to 0.87 (range of subscales)<br>The split-half coefficient α of 0.87 and 0.91
Kim, Kim & Boren 2008<br>Google Scholar citations: 49<br>Article quality rating: 11/15	Kim Alliance Scale Revised (KAS-R)	Revised 16-item questionnaire to measure the quality of the therapeutic alliance from the patient's perspective:<br>• 4 subscales: 1) collaboration, 2) integration, 3) empowerment, and 4) communication	USA<br>n = 601 patients from 2 outpatient clinics serving military family members and retirees	Convergent validity<br>Factor analysis: 4 factors<br>Cronbach's α = 0.89 (overall scale); α = 0.75 to 0.80 (range of subscales)

Comments	Name	Description	Setting & sample	Psychometrics
Marco, Buderer & Thum 2005<br><br>Google Scholar citations: 23<br><br>Article quality rating: 7/15	Questionnaire (no name specified)	18-item questionnaire to measure the opinions of family members of deceased patients around end-of-life care:<br>• Themes include: overall care, nursing care, physician care, pastoral care, pain management, facilities, communication, advanced directives, facilities, and ancillary services	USA<br><br>n = 969 family members of deceased patients	Not reported
Bernal et al. 2007<br><br>Google Scholar citations: 19<br><br>Article quality rating: 7.5/15	Questionnaire (no name specified)	Revised 17-item questionnaire to measure the opinions of family members of deceased patients around end-of-life care:<br>• Questions measure the care and information provided by nurses, doctors, and chaplains; the adequacy of the hospital facilities; and appropriate use of advance directives<br>• 8-items are related to end-of-life care; the remaining 9-items are demographics	USA<br><br>n = 165 family members of deceased patients	Not reported

Comments	Name	Description	Setting & sample	Psychometrics
**Shared Decision Making/Involved with Decision Making**				
Bennett et al. 2010<br><br>Google Scholar citations: 44<br><br>Article quality rating: 10/15	Preparation for Decision Making (PrepDM)	11-item questionnaire to assess how useful a decision aid or other support intervention is at preparing patients to communicate with their practitioner.	Canada<br><br>n = 400 orthopaedic patients from 5 patient groups (spinal stenosis, knee osteoarthritis, herniated disc, chronic low back pain, hip osteoarthritis)	Construct validity<br><br>Factor analysis: unidimensional<br><br>Item Response Theory: Used on unidimensional measures; items = 2.12 to 3.80 to show that the discrimination values for the 10 items were excellent<br><br>Cronbach's α = 0.92 to 0.96 (range of subscales).<br><br>Item-total correlations = 0.75 to 0.81 (range of subscales)
Deber, Krartschmer & Irvine 1996<br><br>Google Scholar citations: 604<br><br>Article quality rating: 9/15	Problem-Solving Decision-Making Scale (PSDM)	Questionnaire to measure problem solving and decision making:<br>• 2 subscales: 1) problem solving and 2) decision-making<br>• PSDM uses 3 brief vignettes (a morbidity, mortality, and quality of life vignette) and asks respondents to answer questions<br><br>PSDM Scale is 1 of 4 sections in the larger Health Care Decisions Survey.	Canada<br><br>n = 300 cardiovascular patients	Factor analysis: 2 factors<br><br>Cronbach's α = 0.71 to 0.90 (range of subscales)

Comments	Name	Description	Setting & sample	Psychometrics
Elwyn et al. 2003<br>Google Scholar citations: 346<br>Article quality rating: 10/15	Observing Patient Involvement (OPTION)	12-item questionnaire to measure the extent to which healthcare professionals involve patients in decisions.	United Kingdom<br>n = 21 general practitioners participated in 186 audiotaped patient consultations	Content validity<br>Factor analysis: unidimensional<br>Cronbach's α = 0.79 (overall scale)<br>Inter-rater agreement = 0.66 (Cohen's kappa)<br>Inter-rater intraclass correlation coefficient = 0.62<br>Inter-rater reliability for 5 consultations have a coefficient of 0.68 (two raters)<br>Test-retest data, the intra-rater reliability generalizability coefficient was 0.66
Elwyn et al. 2013<br>Google Scholar citations: 34<br>Article quality rating: 9/15	CollaboRATE	3-item questionnaire to measure shared decision making in clinical encounters:<br>• 1) How much effort was made to help you understand your health issues?<br>• 2) How much effort was made to listen to the things that matter most to you about your health issues?<br>• 3) How much effort was made to include what matters most to you in choosing what to do next?	USA<br>Stages 1 and 2: n = 27 interview participants recruited from public areas of a Medical Center<br>Stage 3: n = 30 patients recruited immediately after clinic appointments	Content validity

Comments	Name	Description	Setting & sample	Psychometrics
Heggland et al. 2012<br>Google Scholar citations: 1<br>Article quality rating: 14/15	Patient Participation in Decision-making in Surgical Treatment	16-item questionnaire to measure patient participation in surgical treatment decision-making:<br>• 4 subscales: 1) information dissemination, 2) formulation of options, 3) integration of information, and 4) control	Norway<br>n = 451 physicians and nurses on 6 surgical units	Content, convergent, and discriminant validity<br>Factor analysis: 4 factors<br>Cronbach's α = 0.66 to 0.81 (range of subscale)
Lerman et al. 1990<br>Google Scholar citations: 267<br>Article quality rating: 9/15	Perceived Involvement in Care (PICS)	13-item questionnaire to measure patients' perceived involvement in care:<br>• 3 subscales: 1) doctor facilitation, 2) patient physician information exchange, and 3) patient decision making	USA<br>n = 131 patients recruited from a primary care office	Factor analysis: 3 factors<br>Cronbach's α = 0.73 (overall scale)
Martin, Di Matteo & Lepper 2001<br>Google Scholar citations: 51<br>Article quality rating: 11/15	Facilitation of Patient Involvement Scale (FPI)	9-item questionnaire to measure the degree in which patients perceive that their physicians encourage their involvement in care.	Canada<br>Study 1: n = 236 university faculty and staff members<br>Study 2: n = 338 members of an alumni association<br>Study 3: n = 333 school district faculty and staff<br>Study 4: n = 44 undergraduate students<br>Study 5: n = 84 dental office patients	Convergent, criterion, and discriminant validity<br>Factor analysis: unidimensional<br>Cronbach's α = 0.89 to 0.93 (range from different studies)<br>Test-retest reliability > 0.85

Comments	Name	Description	Setting & sample	Psychometrics
Shields et al. 2005<br>Google Scholar citations: 44<br>Article quality rating: 8/15	Rochester Participatory Decision-Making Scale (RPAD)	9-item questionnaire to measure collaborative decision making between patients and physicians.	USA<br>3 sets of participants:<br>n = 100 primary care physicians (internists and family physicians)<br>n = 5 standardized patients (constructed to mimic typical primary care patients)<br>n = 50 patients from each physician's office	Convergent validity<br>Internal consistency coefficient = 0.72<br>Reliability (Spearman-Brown prophecy formula) = 0.53 (overall scale)
Simon et al. 2006<br>Google Scholar citations: 85<br>Article quality rating: 12/15<br>Kriston et al. 2010<br>Google Scholar citations: 131<br>Article quality rating: 11/15	Shared Decision Making Questionnaire (SDM-Q)<br>Shared Decision Making Questionnaire (SDM-Q-9)	11-item questionnaire to measure shared decision making.<br>Revised 9-item questionnaire measuring shared decision making.	Germany<br>n = 675 patients from different medical fields (depression, urology, anaesthesia, gynecology, and general practice)<br>Germany<br>n = 2,351 primary care patients	Construct validity<br>Cronbach's α = 0.77 (overall scale)<br>Factor analysis: unidimensional<br>Cronbach's α = 0.94 (overall scale)

Comments	Name	Description	Setting & sample	Psychometrics
**Satisfaction with Decision/Conflict With Decision**				
Holmes-Rovner et al. 1996<br>Google Scholar citations: 336<br>Article quality rating: 8/15	Satisfaction with Decision (SWD)	6-item questionnaire to assess patients' satisfaction with health care decisions.	USA<br>Pilot study: n = 120 women recruited from university faculty and staff<br>Study 2: n = 252 women recruited through the local press	Discriminant and convergent validity<br>Cronbach's α = 0.86 (overall scale)
Légaré et al. 2010<br>Google Scholar citations: 66<br>Article quality rating: 6/15	Sure of myself, Understand information, Risk-benefit ratio, Encouragement (SURE)	4-item screening test for decisional conflict in patients:<br>• 2 factors: 3 items (certainty, knowledge, and value) load onto one factor; the fourth item (support) loads onto the second factor	USA<br>n = 123 French-speaking pregnant women considering prenatal screening for Down syndrome<br>n = 1474 English-speaking patients referred to watch condition-specific video decision aids	Concurrent and construct validity<br>Factor analysis: 2 factors<br>Cronbach's α = 0.54 (French speaking women); and α = 0.65 (English-speaking women)
O'Connor 1995<br>Google Scholar citations: 1098<br>Article quality rating: 7/15	Decisional Conflict Scale (DCS)	16-item questionnaire to measure perceptions of effective decision making:<br>• 3 subscales: 1) uncertainty, 2) effective-decision-making, and 3) factors-contributing to uncertainty	Canada<br>n = 909 patients with cardiac and respiratory disorders, students, and health employees	Construct and discriminant validity<br>Cronbach's α = 0.78 to 0.92 (range of subscales); α = 0.58 to 0.92 (range of subscales)<br>Test-retest reliability coefficient = 0.81
**Communication**				

Comments	Name	Description	Setting & sample	Psychometrics
Campbell et al. 2007<br>Google Scholar citations: 47<br>Article quality rating: 8/15	Questionnaire (no name specified)	19-item questionnaire to measure the physician-patient communication skills in practicing physicians:<br>• 2 subscales: 1) process of communication and 2) content of communication<br>• 2 versions of the questionnaires: a physician version and a patient version	Canada<br>n = 1,845 patient/ physician dyads were surveyed<br>Study was conducted with family doctors and specialists; data provided from 25 visits	Factor analysis: 2 factors for both physicians and patients<br>Cronbach's α = 0.70 (physicians); α = 0.69 (patients)
Cegala, Thoesen Coleman & Turner 1998<br>Google Scholar citations: 90<br>Article quality rating: 8/15	Medical Communication Competence Scale (MCCS)	38-item physician questionnaire and 41-item patient questionnaire to measure perceptions of self- and other communication during medical consultations:<br>• 4 subscales for both versions: 1) information giving, 2) information seeking, 3) information verifying, and 4) socio-emotional communication	USA<br>n = 65 physicians and n = 52 patients from different sites	Content validity<br>Cluster analysis determined where the items fit into dimensions of the scale<br>Cronbach's α = 0.75 to 0.90 (range of subscales for physicians' scale); α = 0.76 to 0.92 (range of subscales for patients' scale)
Edwards et al. 2003<br>Google Scholar citations: 112<br>Article quality rating: 11/15	Combined Outcome Measure for Risk Communication and Treatment Decision Making Effectiveness (COMRADE)	20-item questionnaire to measure decision effectiveness:<br>• 2 subscales: 1) risk communication and 2) confidence in the decision	United Kingdom<br>n = 960 patients with known atrial fibrillation, prostatism, menorrhagia or menopausal symptoms	Content and construct validity<br>Factor analysis: 2 factors

Comments	Name	Description	Setting & sample	Psychometrics
Farin, Gramm & Kosiol 2011<br>Google Scholar citations: 23<br>Article quality rating: 12/15	Communication preferences of patients with chronic illness' (KOPRA)	32-item questionnaire to measure the communication preferences of chronically ill patients:<br>• 4 subscales: 1) patient participation and patient orientation, 2) effective and open communication, 3) emotionally supportive communication, and 4) communication about personal circumstances	Germany<br>n = 472 patients with chronic back pain or chronic ischemic heart disease who were undergoing inpatient rehabilitation<br>n = 333 surveyed on communication preferences with physician<br>n = 139 tested the applicability with nurses and therapists	Factor analysis: 4 factors<br>Scales are unidimensional and fulfill demands for 1-parameter IRT model<br>Cronbach's α = 0.80 to 0.92 (range of subscales)
Stewart et al. 2007<br>Google Scholar citations: 90<br>Article quality rating: 8/15	Interpersonal Processes of Care (IPC)	29-item (full version) and 18-item (short version) questionnaires to measure aspects of communication, decision-making, and interpersonal style:<br>• Full version has 12 first order subscales and 7 second order subscales<br>• Short version has 7 subscales	USA<br>n = 1,664 patients in adult general medicine care	Content validity<br>Extensive factor model testing determined the appropriate fit<br>Cronbach's α = 0.65 to 0.90 (range of subscales)
**Empowerment and Empathy**				
Gagnon et al. 2006<br>Google Scholar citations: 40<br>Article quality rating: 11/15	The Health Care Empowerment Questionnaire (HCEQ)	10-item questionnaire to measure the degree of individual empowerment in relation to personal health care and services:<br>• 3 subscales: 1) involvement in decisions, 2) degree of control, and 3) involvement in interactions	Canada<br>n = 873 patients who had contact with health professionals during the past 6 months	Construct, convergent, and discriminant validity<br>Factor analysis: 3 factors<br>Cronbach's α = 0.83 (overall scale)<br>Intraclass correlation coefficients = .70

Comments	Name	Description	Setting & sample	Psychometrics
Mercer et al. 2004<br><br>Google Scholar citations: 222<br><br>Article quality rating: 10/15	Consultation and Relational Empathy (CARE)	10-item questionnaire to measure empathy during the consultation process.	United Kingdom<br><br>n = 43 patient interviews<br><br>n = 20 general practitioner colleagues associated with departments of general practice and primary care	Content and concurrent validity<br><br>Cronbach's α = 0.92 (overall scale)

Principle 3: Geographic Coverage and Rostering

Primary care network structures in place

Author	Name	Description	Setting & Sample	Psychometrics
Birnberg et al. 2011<br>Google Scholar citations: 27<br>Article quality rating: 12/15	Safety Net Medical Home Scale (SNMHS)	57-item questionnaire to measure patient-centered medical home characteristics:<br>• 5 subscales: 1) access to care and communication with patients and other providers; 2) patient tracking and registry; 3) care management, test and referral tracking; 4) quality improvement; and 5) external coordination	USA<br>n = 65 safety-net clinics (completed by health center leadership)	Convergent validity<br>Cronbach's α = 0.84 (overall scale); α = 0.60 to 0.89 (range of subscales)
Cassady et al. 2000<br>Google Scholar citations: 143<br>Article quality rating: 8/15	Primary Care Assessment Tool - Child Edition (PCAT-CE)	26-item questionnaire to evaluate the attainment of key characteristics of primary care services for children and youth:<br>• 5 subscales: 1) longitudinally-relationship, 2) first-contact accessibility, 3) comprehensiveness-services available, 4) comprehensiveness-services provided, and 5) coordination<br>Adolescent and adult versions were developed.	USA<br>n = 450 parents and guardians of children ≤ 18	Content and construct validity<br>Cronbach's α = 0.40 to 0.86 (range of subscales)

Author	Name	Description	Setting & Sample	Psychometrics
Cooley et al. 2003<br>Google Scholar citations: 79<br>Article quality rating: 10/15	Medical Home Index – Long Version (MHI-LV)	25-item questionnaire to assess primary care pediatric practices:<br>• 6 subscales: 1) organizational capacity, 2) chronic condition management, 3) care coordination, 4) community outreach, 5) data management, and 6) quality improvement<br>• The MHI was also extracted in domain 5.1<br>Adult and child versions were developed.	USA<br>n = 43 primary care pediatric practice sites	Construct validity<br>Cronbach's α = 0.96 (overall scale); α = 0.81 to 0.92 (range of subscales)<br>Kappa range of 0.65 or better (for 80% of themes)<br>Intraclass correlations coefficients = 0.99
Centre for Medical Home Improvement (CMHI) website 2006 (Grey Literature)<br>Google Scholar citations: 0<br>Article quality rating: N/A	Medical Home Index – Short Version (MHI-SV)	14-item questionnaire to measure the degree to which a practice has achieved components of a medical home:<br>• Same six subscales as the MHI-LV<br>Adult and child versions were developed.	Not reported	The short version of the MHI did not go through the same psychometric testing as the long version, but it was developed through the same statistical process
Flocke 1997<br>Google Scholar citations: 205<br>Article quality rating: 9/15	Components of Primary Care Index (CPCI)	19-item questionnaire to measure components of primary care from the perspective of the patient:<br>• 4 subscales: 1) patient preference for their regular physician, 2) interpersonal communication, 3) accumulated knowledge of patient, and 4) coordination of care	USA<br>n = 2,899 patients who visited 138 family physicians' offices	Content validity<br>Factor analysis: 4 factors<br>Cronbach's α = 0.68 to 0.79 (range of subscales)

Author	Name	Description	Setting & Sample	Psychometrics
Friedberg et al. 2008<br><br>Google Scholar citations: 82<br><br>Article quality rating: 9/15	Questionnaire (no name specified)	13-item questionnaire to assess the structural capabilities among primary care practice:<br>• 4 key subscales of structural capabilities: 1) patient assistance and reminders, 2) culture of quality, 3) enhanced access, and 4) electronic health records	USA<br><br>n = 308 physician practitioners	Not reported
Nikbakht-Van De Sande et al. 2005<br><br>Google Scholar citations: 26<br><br>Article quality rating: 9/15	Questionnaire (no name specified)	200-item questionnaire to evaluate structure, process, and outcome measures of palliative care networks:<br>• Items related to structure: demographic characteristics, history, start, developmental stage, resources, and participating organizations of the network<br>• Items concerning process: organization and management, cooperation, and external relationships<br>• Items related to outcome: shared objectives and perceptions, the demand for care, the quality of cooperation, the improvement of care services, achieved agreements, results associated with individual participants, patients and organizations, expertise, and funding	The Netherlands<br><br>n = 92 managers and care providers of 8 palliative care networks	Not reported

Author	Name	Description	Setting & Sample	Psychometrics
Rittenhouse et al. 2008<br>Google Scholar citations: 144<br>Article quality rating: 7/15	Questionnaire (no name specified)	Questionnaire to measure infrastructure components of the medical home:<br>• 4 subscales: 1) physician-directed medical practice, 2) care coordination, 3) quality and safety, and 4) enhanced access component	USA<br>n = 291 medical groups	Not reported
Rodrigues et al. 2014<br>Google Scholar citations: 3<br>Article quality rating: 15/15	Instrumento de Avaliação da Coordenação das RAS pela APS (COPAS)<br>The Tool for Assessment of the Coordination of Integrated Health Service Delivery Networks by the Primary Health Care	78-item questionnaire to evaluate the coordination of primary care networks on the degree of integration of healthcare networks:<br>• 5 subscales: 1) population, 2) primary care, 3) logistics system, 4) support system, and 5) management system	Brazil<br>n = 150 random sample of healthcare professionals	Content, convergent, and discriminant validity<br>Cronbach's α = 0.66 to 0.87 (range of subscales)

Principle 4: Standardized Care Delivery through Interprofessional Teams

Team effectiveness

Author	Name	Description	Setting & Sample	Psychometrics
		**Interprofessional Teams**		
Amundson 2005<br>Google Scholar citations: 32<br>Article quality rating: 11/15	Team Effectiveness Scale	7-item team member and 5-item supervisor questionnaire to measure team effectiveness:<br>• both versions include team performance indicators (e.g., team efficiency)<br>The Group Emotional Competence Scale (66-items) was also used in the study and significantly predicted team effectiveness.	USA<br>n = 85 individuals from 20 healthcare and human services teams from 11 medical and social services centres	Cronbach's α = 0.89 (team scale); α = 0.58 (supervisor's scale)
Bateman, Wilson & Bingham 2002<br>Google Scholar citations: 43<br>Article quality rating: 10/15	Team Effectiveness Audit Tool	44-item questionnaire to measure effective teams:<br>• 4 subscales: 1) effectiveness of team outputs, 2) team identity/team synergy, 3) clarity of performance, and 4) team role clarity<br>• Based on 6 core themes: 1) team synergy, 2) performance objectives, 3) skills, 4) use of resources, 5) innovation, and 6) quality	United Kingdom<br>n = 400 participants across 37 health and social teams in public sector organizations	Factor analysis: 4 factors<br>Cronbach's α = 0.98 (overall scale)<br>Inter-item reliability = 0.97 to 0.98 (range of subscales)

Author	Name	Description	Setting & Sample	Psychometrics
Cramm & Nieboer 2011<br>Google Scholar citations: 7<br>Article quality rating: 11/15	Questionnaire (no name specified)	10-item scale to measure interprofessional stroke team functioning:<br>• examines communication, planning, support, and decision-making processes<br>The interprofessional stroke team functioning items (10-items) are a part of a larger 33-item questionnaire to assess professional's views on interprofessional stroke team functioning.	The Netherlands<br>n = 558 professionals within 34 stroke teams at 12 hospitals, 16 nursing homes, 6 rehabilitation centres in 9 towns	Face validity<br>Cronbach's α = 0.81 (interprofessional stroke team functioning scale)
Schroder et al. 2011<br>Google Scholar citations: 40<br>Article quality rating: 12/15	Collaborative Practice Assessment Tool (CPAT)	56-item questionnaire for interprofessional teams to measure their collaborative practice:<br>• 8 subscales: 1) mission and goals, 2) relationships, 3) leadership, 4) role responsibilities and autonomy, 5) communication, 6) decision-making and conflict management, 7) community linkages and coordination, and 8) patient involvement	Canada<br>n = 111 practitioners completed the CPAT during final testing of the scale	Content validity<br>Factor analysis: 8 factors<br>Cronbach's α = 0.67 to 0.89 (range of subscales)

Author	Name	Description	Setting & Sample	Psychometrics
Shortell et al. 1991<br>Google Scholar citations: 437<br>Article quality rating: 14/15	ICU nurse-physician questionnaire	18-item questionnaire to measure perceived unit effectiveness of ICU nurses and physicians:<br>• perceived unit effectiveness was measured by looking at the correlations of the other questionnaire items and 3 “outcome” measures of effectiveness: 1) absolute technical quality of care, 2) meeting family members’ needs, and 3) nurse turnover<br>• 2 versions of the questionnaire: nurse-long and physician-long<br>The perceived effectiveness items (18-items) are a part of a larger questionnaire (218-items) to measure clinician perceptions of collaborative interactions.	USA<br>Pilot test: n = 134 nurses and n = 53 physicians from 5 ICUs at 4 hospitals<br>Revised questionnaire: n = 1,418 nurses, n = 790 physicians, n = 111 unit ward clerks, and n = 221 top management team members of 42 medical/ surgical ICUs	Perceived unit effectiveness Cronbach’s α = 0.75<br>ANOVA determined the data could be aggregated to the unit level
Smits et al. 2003<br>Google Scholar citations: 28<br>Article quality rating: 11/15	Patient-focused rehabilitation cohesiveness scale	20-item questionnaire to measure rehabilitation team functioning:<br>• 3 subscales: 1) effort, 2) teamwork, and 3) perceived effectiveness	USA<br>In-patient rehabilitation unit teams<br>50 teams participated; n = 650 health care providers	Scale items based on validated scales and pilot-tested for this population<br>Cronbach’s α = 0.96 (overall scale)
Temkin-Greener et al. 2004<br>Google Scholar citations: 116<br>Article quality rating: 11/15	Questionnaire (no name specified)	59-item questionnaire to identify team effectiveness as an outcome of team performance in community-based long-term care:<br>• 6 subscales: 1) perceived team effectiveness, 2) leadership, 3) coordination, 4) communication, 5) conflict management, and 6)team cohesion	USA<br>26 All-Inclusive Care for the Elderly (PACE) programs; n = 1,200 participants (health care providers and allied health)	Face, content, and construct validity<br>Cronbach’s α = > 0.73 (overall scale)<br>ANOVAs to assess variance within and between teams

Author	Name	Description	Setting & Sample	Psychometrics
Temkin-Greener et al. 2009<br>Google Scholar citations: 28<br>Article quality rating: 14/15	Nursing-home Work Environment and Performance Team Survey	56-item questionnaire to measure predictors of perceived work effectiveness in nursing homes:<br>• 5 subscales: 1) leadership, 2) communication/ coordination, 3) conflict management, 4) work group cohesion, and 5) perceived work effectiveness<br>• 2 control variables measure workplace conditions and resources and staffing<br>The Nursing-home Work Environment and Performance Team Survey was developed based on the 2004 Temkin-Greener et al. questionnaire.	USA<br>n = 7,418 managers and direct care staff (e.g., professionals: physicians, nurses and paraprofessionals: Certified Nursing Attendants) of 162 nursing homes	Face and content validity<br>Convergent-divergent validity assessed with Pearson correlation coefficients<br>Construct validity<br>Factor analysis: unidimensional<br>Cronbach's α = 0.78 to 0.89 (range of subscales for professionals); α = 0.73 (control variables)<br>Cronbach's α = 0.74 to 0.86 (range of subscales for para-professionals); α = 0.71 to 0.74 (range of subscales for control variables)
Undre et al. 2006<br>Google Scholar citations: 123<br>Article quality rating: 9.5/15	Observational Teamwork Assessment for Surgery (OTAS)	A 2-element practical method to assess teamwork specific to the general surgical environment includes:<br>• A task checklist completed by a surgical observer to capture task completion<br>• An assessment of team behavior, completed by a post-doctoral psychologist, on five subscales: 1) coordination, 2) extracting information, 3) using authority, 4) supporting others, and 5) assessing capabilities	United Kingdom<br>n = 50 general surgery operations from one operating theater which include anesthetists, nurses, surgeons, and operating theater assistants	ANOVAs to access differences on behaviours and phases<br>Spearman's rho: Positive correlation coefficients between task completion and team behaviours

Author	Name	Description	Setting & Sample	Psychometrics
Vinokur-Kaplan 1995<br><br>Google Scholar citations: 144<br><br>Article quality rating: 11.5/15	Questionnaire (no name specified)	30-item questionnaire based on Hackman's Conceptual Model of Team Effectiveness:<br>• 5 independent variable: 1) group size, 2) task clarity, 3) feelings of influence on teammates, 4) consultation available, and 5) environmental support<br>• 3 mediating variables: 1) members presence at meetings, 2) nterdisciplinary collaboration, and 3) group interdependence<br>• 4 dependent variables: 1) standards met, 2) cohesion, 3) individual well-being, and 4) team effectiveness<br>• 3-stages of analysis: individual-, group-, and intergroup-level	USA<br><br>n = 98 mental health professionals from 15 teams in 3 public psychiatric hospitals	Convergent validity<br><br>Cronbach's α = 0.65 to 0.89 (range of subscales)
**Virtual Teams (Non-Health care)**				
Lurey & Raisinghani 2001<br><br>Google Scholar citations: 559<br><br>Article quality rating: 12/15	Virtual Teams Survey	8-item questionnaire to measure virtual team performance:<br>• 2 main criteria of team effectiveness were established: 1) teams' abilities to perform their work assignments and 2) team members' levels of satisfaction while working with their virtual teams<br><br>The team performance items (8-items) are a part of a larger scale in a larger questionnaire on virtual teams (82-items).	USA, Europe, Asia<br><br>n = 67 individuals from 12 virtual teams from 8 companies including the high technology, agriculture, and professional services industries	Cronbach's α = 0.82 (overall team performance scale)<br><br>Correlation between performance and satisfaction = 0.73

Author	Name	Description	Setting & Sample	Psychometrics
Staples & Webster 2007<br>Google Scholar citations: 85<br>Article quality rating: 13/15	Self-efficacy for teamwork measure	86-item questionnaire to measure self-efficacy for teamwork in traditional and virtual teams:<br>• 11 subscales: 1) modeling by team members, 2) modeling by team leaders, 3) coaching by team members, 4) coaching by team leaders, 5) organizational practices and training, 6) self-efficacy for teamwork, 7) coping ability, 8) individual performance, 9) intention to remain on the team, 10) team performance, and 11) satisfaction with the team	Canada<br>n = 493 team members from high technology, consulting, and manufacturing	Structural equation modelling used to determine discriminant validity and internal consistency<br>Internal consistency = 0.88 to 0.96 (range of subscales)<br>Cronbach's α = 0.78 to 0.96 (range of subscales)
**Grey Literature**				
Hepburn, Tsukuda & Fasser 1998<br>Google Scholar citations: 0<br>Article quality rating: N/A	Team Skills Scale	17-item questionnaire to measure perceived levels of team skills related to geriatric care:<br>• Questions measure: interpersonal skills, discipline specific skills, and geriatric care skills<br>• The questionnaire was developed to measure changes in the skills of nurses before and after a team training intervention	Not reported	Not reported

Author	Name	Description	Setting & Sample	Psychometrics
Hyer, Heinemann & Fulmer 2002<br>Google Scholar citations: 29<br>Article quality rating: 5/15	Team Skills Scale (reporting psychometrics)	Same as above. This book chapter reports psychometrics.	Not reported	Content and face validity<br>Factor analysis: unidimensional<br>Chronbach's α = 0.94 (overall scale)<br>Item-to-total scale correlations = 0.58 to 0.78

Use of shared clinical pathways across the continuum of health care and geography; and Individualization of care pathways for patients with co-morbidities

Author	Name	Description	Setting & Sample	Psychometrics
**4.2 Use of Shared Clinical Pathways across the Continuum of Hear Care and Geography**				
Ainsworth & Buchan 2012<br>Google Scholar citations: 10<br>Quality rating: 9.5/15	Collaborative Online Care Pathway Investigation Tool (COCPIT)	Instrument to conduct a care pathway variance analysis:<br>• 3 components: 1) data management framework, providing access to individual medical records; 2) visual editor for designing integrated care pathways; and 3) analysis and visualization component for care pathway variance analysis	United Kingdom<br>Salford Integrated Record database for patients with chronic kidney disease<br>Stroke patients at Salford Royal Foundation Trust	Not reported
Vanhaecht et al. 2007<br>Google Scholar citations: 35<br>Quality rating: 11.5/15	Care Process Self-Evaluation Tool (CPSET)	29-item questionnaire to measure how a clinical pathway influences the process of patient care:<br>• 5 subscales: 1) patient-focused organization, 2) coordination of the care process, 3) communication with patients and family, 4) collaboration with primary care, and 5) monitoring/ follow-up of the care process	United Kingdom<br>n = 6 hospitals from the Belgian-Dutch Clinical Pathway Network	Face, content, construct, and criterion validity<br>Cronbach's α = 0.78 to 0.93 (range of subscales)<br>Intraclass correlation coefficients = 0.28 to 0.70 (range of subscales)
Van Houdt et al. 2013<br>Google Scholar citations: 17<br>Quality rating: 11/15	Questionnaire (no name specified)	38-item questionnaire to assess the effect of a care pathway using patient perceived quality indicators for those with prostate cancer:<br>• 5 subscales: 1) communication and coordination between caregivers, 2) information towards patient, 3) consultation of specialists, 4) patient outcomes, and 5) general health	Belgium<br>n = 92 patients treated with radical prostatectomy from the Bruges region	Face and content validity

Author	Name	Description	Setting & Sample	Psychometrics
Wagner et al. 2014<br>Google Scholar citations: 14<br>Quality rating: 11.5/15	Checklist (no name specified)	39-item checklist to assess the implementation of quality management activities across 4 different care pathways:<br>• 4 subscales: 1) quality improvement, 2) evidence based practice, 3) patient safety strategies, and 4) organizational structure of the pathway<br>• The number of items per subscale differs by care pathway, as some items are disease specific; 15 items were applicable across all 4 care pathways	Europe<br>n = 74 hospitals in France, Poland, Turkey, Portugal, Spain, Germany and Czech Republic with >130 beds who delivered care for acute myocardial infarction, hip fracture, stroke, or deliveries	Cronbach's α = 0.46 to 0.86 (range of subscales)
Whittle et al. 2004<br>Google Scholar citations: 24<br>Quality rating: 12/15	Integrated Care Pathways Assessment Tool (ICPAT)	39-item instrument to evaluate the quality of integrated care pathways:<br>• 2 forms are used by appraisers under 6 subscales (2 subscales under the first form and 4 subscales under the second form)<br>• Form 1: 1) face validity, 2) documentation Form 2: 3) development process, 4) implementation process, 5) maintenance, 6) role of the organization	United Kingdom<br>n = 68 participants from 25 National Health Service trusts<br>Evaluated anonymous care pathways already in place for total hip replacement, myocardial infarction, leg ulcer and mental health care	Construct validity<br>Cronbach's α = 0.77 to 0.96 (range of subscales)<br>Intraclass correlation coefficients = 0.63 to 0.99 (range of subscales)
**4.3 Individualization of care pathways for patients with co-morbidities**				
Glasgow et al. 2005<br>Google Scholar citations: 407<br>Article quality rating: 13/15	Patient Assessment of Chronic Illness Care (PACIC)	20-item questionnaire to measure if patient's receipt of clinical services are consistent with the Chronic Care Model:<br>• 5 subscales: 1) patient activation, 2) delivery system design/decision support, 3) goal setting, 4) problem solving/contextual counselling, and 5) follow-up/coordination	USA<br>n = 283 enrollees age 50 or older receiving care from 7 primary care clinics within Group Health Cooperative	Face, content, and concurrent validity<br>Test-retest reliability over a three-month interval, r = 0.58

Author	Name	Description	Setting & Sample	Psychometrics
Drewes et al. 2012<br>Google Scholar citations: 15<br>Article quality rating: 14/15	Patient Assessment of Chronic Illness Care + (PACIC+)	Revised 26-item patient report instrument to measure the chronic care management experience of those with diabetes:<br>• Includes all subscales of PACIC<br>• Additional items for multidisciplinary team functioning	The Netherlands<br>n = 1,941 diabetes patients	Cronbach's α = 0.92 (PACIC); α = 0.91 (PACIC+)
**Grey Literature**				
Wagner, Austin & Von Korff 1996<br>Google Scholar citations: 2,290<br>Article quality rating: 12/15	Essential elements for creating a care pathway for those with multiple chronic conditions (no name specified)	Provides components of high-quality chronic illness care:<br>• 4 essential elements of successful programs: 1) collaborative problem definition, 2) targeting, goal setting and planning, 3) a continuum of self-management training and support services, and 4) active and sustained follow-up	Not reported	Not reported

Principle 5: Performance Management

Performance measurement indicators and tools in place and Clinical outcomes being measured

Author	Name	Description	Setting & Sample	Psychometrics
Andrade, Vaitsman & Otávio Farias 2010<br>Google Scholar citations: 1<br>Article quality rating: 8/15	Índice de Responsividade do Serviço (IRS)<br>Health Service Responsiveness Index (SRI)	160-item questionnaire to measure patients' positive or negative perceptions in 2 subscales of responsiveness:<br>• patient orientation: components that influence patient satisfaction, but are not directly connected with health care: agility, social support, facilities and choice<br>• personal respect: dignity, confidentiality, autonomy	Brazil<br>n = 298 Patient data at the Evandro Chagas Research Institute, a unit of the Oswaldo Cruz Foundation, Rio de Janeiro	Not reported
Cooley et al. 2003<br>Google Scholar Citations: 79<br>Quality Rating: 10/15	Medical Home Index (MHI)	See domain 3.1 for questionnaire details.		
Centre for Medical Home Improvement (CMHI) website 2006 (Grey Literature)<br>Google Scholar citations: 0<br>Article quality rating: N/A	Medical Home Index – Short Version (MHI-SV)	See domain 3.1 for questionnaire details.		

Data tracked and shared with stakeholders

No unique tools were found for this indicator domain.

Principle 6: Information Systems

Shared information systems across sectors

No unique tools were found for this indicator domain.

Shared patient electronic charts across continuum of care assessable to patients

Author	Name	Description	Setting & Sample	Psychometrics
Chou et al. 2010<br>Google Scholar citations: 8<br>Quality rating: 6/15	Promoting patient-centered preventative care using a wellness portal: preliminary findings	A mixed methods approach (questionnaire) to evaluate data from a field test of a wellness portal for patients in primary care settings:<br>• Surveyed via structured (4-point Likert scale) and open ended questions<br>• Frequency statistics and content analysis used<br>• Ease of use, patient perceptions, and potential impact	USA<br>n = 30 Patients from 2 practices in a Physicians Resource/Research Network	Not reported

Data collected is used for service planning

Author	Name	Description	Setting & Sample	Psychometrics
Wilkinson & McCarthy 2007<br>Google Scholar citations: 4<br>Quality Rating: 9/15	Use of comparative data for integrated cancer services	Questionnaire to measure how much individuals working in cancer networks used 7 data sets available in England.	England<br>n = 29 cancer networks teams with n = 68 individual participants	Not reported

Principle 7: Organizational Culture and Leadership

Organizational goals and objectives aligned across sectors

Author	Name	Description	Setting & Sample	Psychometrics
Cameron & Quinn 2005<br>Google Scholar citations: 4,419<br>Quality Rating: 6/15	Organizational Culture Assessment Instrument (OCAI)	24-item questionnaire to measure organizational culture:<br>• 6 domains: 1) dominant characteristics, 2) organizational leadership, 3) management of employees, 4) organization glue, 5) strategic emphases, and 6) criteria of success	USA	Discriminant, convergent, and concurrent validity<br>Cronbach's α = 0.67 to 0.83 (range of subscales)

8. Physician Integration

Physician integration within care teams and across sectors

Author	Name	Description	Setting & Sample	Psychometrics
Chesluk et al. 2012<br>Google Scholar Citations: 14<br>Quality Rating: 7/15	Team Effectiveness Assessment Model (TEAM)	A 4-step process for physicians to assess their performance in interprofessional collaborative practice:<br>• 4 tools: 1) criteria for identifying interprofessional team, 2) physician self-assessment survey (29-items), 3) rater assessment of physician (29-items), 4) guided debrief (17-items)<br>• Quantitative and qualitative feedback<br>• Guidance on how to analyze and use feedback received	USA<br>n= 25 hospitalists	Not reported
Dynan, Bazzoli & Burns 1998<br>Google Scholar Citations: 58<br>Quality Rating: 9/15	Survey Instrument (no name specified)	44-item questionnaire to measure and compare the level of integration across physician-hospital models and structures and processes that facilitate physician-hospital integration:<br>• 6 subscales: 1) administrative and practice management services, 2) physician financial risk sharing, 3) joint ventures to create new services, 4) computer linkages, 5) physician involvement in strategic planning, and 6) salaried physician arrangements	USA<br>n = 573 hospitals	Factor analysis: 6 factors

Author	Name	Description	Setting & Sample	Psychometrics
Milette, Hébert & Veil 2005<br>Google Scholar Citations: 6<br>Quality Rating: 8/15	Two questionnaires (no name specified)	Cross-sectional survey; 2 questionnaires administered 6 months apart to measure family physicians' perceptions of integrated service delivery (ISD) networks:<br>• Baseline survey: perceptions of ISD networks, perceived role, and receptivity to new case management role<br>• 6 month follow-up survey: perceptions of ISD networks and challenges to case management	Canada<br>n = 124 family physicians	Not reported
Smits et al. 2003<br>Google Scholar Citations: 28<br>Quality Rating: 11/15	Physician Support Scale	9-item questionnaire to assess change in team effectiveness:<br>• Terminology was changed to better reflect the setting (team vs group and attending physician vs leader)<br>• True/false questions to be completed about the attending physician by other health care providers	USA<br>In-patient rehabilitation unit teams<br>50 teams participated; n = 650 health care providers	Cronbach's α = 0.82 (overall scale)
Smits et al. 2003<br>Google Scholar Citations: 28<br>Quality Rating: 11/15	Physician Involvement	9-item questionnaire completed by other team members to assess attending physician effort in activities that were likely to impact team effectiveness.	USA<br>In-patient rehabilitation unit teams<br>50 teams participated; n = 650 health care providers	Not reported

Author	Name	Description	Setting & Sample	Psychometrics
Southern, Appleby & Young 2001<br><br>Google Scholar Citations: 1<br><br>Quality Rating: 6/15	Questionnaire (no name specified)	114–item questionnaire to measure general practitioners' (GP) perceptions of a well-integrated GP:<br>• 10 subscales: 1) holistic and flexible practice, 2) care coordination, 3) attitudes towards teamwork, 4) community health planning, 5) political linkages, 6) knowledge and education, 7) time and funding, 8) practice organization, 9) information technology, and 10) personal domain/personal attributes	Australia<br><br>n = 208 general practitioners	Not reported

Principle 10: Financial Management

Attainment of goals and objectives are supported by funding and human resource allocation

Author	Name	Description	Setting & Sample	Psychometrics
Bradford et al. 2000<br><br>Google Scholar citations: 2<br><br>Quality rating: 8/15	No name	Questionnaire to assess the effectiveness of resource allocation:<br>• 4 subscales: 1) priority-setting methods, 2) grants-making methods, 3) service-monitoring methods, and 4) outcomes-assessment methods	USA<br><br>n = 133, statewide mail survey of consortia members, including organizational representatives and individuals	Cronbach's α = 0.82 (Priority-setting methods); α = 0.76 (Grants-making methods); α = 0.69 (Service-monitoring methods); α = 0.68 (Outcomes-assessment methods)

Overall Integration Instruments

Author	Name	Description	Setting & Sample	Psychometrics
Abendstern et al. 2006<br><br>Google Scholar citations: 19<br><br>Article quality rating: 7/15	Questionnaire (no name specified)	Questionnaire to measure structural and process indicators of integration:<br>• Structural features: structural level integration, practice level integration, and specialization<br>• Process features: assessment and care planning, accessibility, person-centered care, and carer involvement	England<br><br>n = 52 professional community teams in dementia care services	Not reported
Bainbridge et al. 2015<br><br>Google Scholar citations: 5<br><br>Article quality rating: 10/15	Health Care Providers (HCP) Integration Survey	60-item questionnaire to measure the system structure and process of care of integrated care systems:<br>• 5 subscales: 1) interdependence, 2) newly created professional activities, 3) flexibility, 4) collective ownership and goals, and 5) reflection on process	Canada<br><br>n = 86 Palliative Care Network healthcare providers	Content validity<br><br>Cronbach's α = 0.92 (overall scale)

Author	Name	Description	Setting & Sample	Psychometrics
Devers et al. 1994<br><br>Google Scholar citations: 149<br><br>Article quality rating: 8/15	Integration measures (no name specified)<br><br>(measures can be used to develop a Systems Integration Scorecard)	49-item questionnaire to measure functional, physician-system, and clinical integration:<br>• 8 areas assess functional integration: 1) culture, 2) strategic planning, 3) human resources, 4) financial management, 5) information systems, 6) support services, 7) quality assurance/quality improvement, and 8) other<br>• 4 areas assess physician-system integration: 1) economic involvement, 2) administrative involvement, 3) group practice formation, and 4) shared accountability<br>• 6 areas assess clinical integration: 1) clinical protocol development, 2) medical records uniformity and accessibility, 3) clinical outcomes data collection and utilization, (4) clinical programming and planning efforts, 5) shared clinical support services, and 6) shared clinical service lines	USA<br><br>Objective measures were collected through a questionnaire of personnel in the system and operating unit offices (e.g., personnel in corporate physician affairs office, staff in physician groups, etc.)	Content validity

Author	Name	Description	Setting & Sample	Psychometrics
Friedman et al. 2014<br><br>Google Scholar citations: 12<br><br>Article quality rating: 7/15	Multi-disciplinary Clinics and Conferences (MDC) Assessment Tool, Version 3.0	Assessment tool to measure maturation of multidisciplinary care:<br>• 9 assessment areas: 1) case planning, 2) physician engagement, 3) treatment team integration, 4) integration of care coordinators, 5) infrastructure, 6) financial, 7) clinical trials, 8) medical records, and 9) quality improvement<br>• The assessment areas are rated on 5 levels; 4 areas use only 3 levels<br>• Respondents were also asked to provide details on what they did to move up a level in the rating scale	USA<br><br>n = 14 National Cancer Institute Community Cancer Centers Program sites	Not reported
Gillies et al. 1993<br><br>Google Scholar citations: 237<br><br>Article quality rating: 11/15	Questionnaire (no name specified)	54-item questionnaire to measure perceived functional integration, physician-system integration, and clinical integration:<br>• 12 subscales: 1) human resources, 2) support services, 3) culture 4) strategic planning, 5) quality assurance, 6) marketing, 7) information systems, 8) financial management – resource allocation, 9) financial management – operating policies, 10) functional integration – average, 11) physician integration, and 12) clinical integration	USA<br><br>n = 933 members of 9 healthcare systems participated including: managers, board members, physicians, non-corporate management, and corporate office-level management	Factor analysis: 12 factors<br><br>Cronbach's α = 0.57 to 0.93 (range of subscales)

**Author**	**Name**	**Description**	**Setting & Sample**	**Psychometrics**
Hébert & Veil 2004<br><br>Google Scholar citations: 73<br><br>Article quality rating: 6/15	PRISMA model implementation scale	20-indicators selected to rate the implementation of integrated service delivery:<br>• 6 mechanisms and tools include: 1) coordination of all organizations involved in delivering health and social services; 2) a single entry point, 3) case management, 4) a single assessment tool with a case-mix classification system, 5) an individualized service plan, and 6) a computerized clinical chart	Canada<br><br>n = 3 areas in Quebec where the PRISMA model was being implemented	Content validity
Nelson et al. 2002<br><br>Google Scholar citations: 430<br><br>Article quality rating: 8/15	Clinical Microsystem Assessment Tool (CMAT)	The CMAT is a tool that assesses how a clinical microsystem compares to the 10 key "success" characteristics of high-performing clinical microsystems:<br>• 1) leadership, 2) organizational support, 3) staff focus, 4) education and training, 5) interdependence, 6) patient focus, 7) community and market focus 8) performance results, 9) process improvement, and 10) information and information technology<br><br>The Nelson et al. 2002 article had only 9 "success" characteristics. We included the 10 characteristics from the updated scale (Johnson 2001).	USA<br><br>n = 20 microsystems representing different components of health systems	Content validity

Author	Name	Description	Setting & Sample	Psychometrics
Ouwens et al. 2007<br><br>Google Scholar citations: 41<br><br>Article quality rating: 7/15	Integrated Care Indicators	8-clinical indicators to measure the quality of integrated care:<br>• 4 areas: 1) multidisciplinary patient care team, 2) integrated care pathway, 3) case management, and 4) patient involvement<br><br>The 8 integrated care indicators were identified along with an additional 23-indicators specific to head and neck cancers.	The Netherlands<br><br>n = 158 patients with head and neck cancers and 15 professionals	Content validity<br><br>Indicators where reliability could be tested had ƙ values of 0.6 or higher
VanDeusen Lukas et al. 2002<br><br>Google Scholar citations: 23<br><br>Article quality rating: 10/15	The Integration Survey	Questionnaire to measure system integration:<br>• 9 subscales represent different staff perspectives: all staff and managers only<br>• All staff scales: 1) leadership, 2) staff cooperation, 3) clinical coordination, 4) service cooperation, 5) alignment,<br>• Manager only scales: 6) shared vision, 7) quality improvement, 8) single standard of care, and 9) manager alignment	USA<br><br>n = 1,042 staff, managers, and clinicians from 5 veteran affairs medical centers	Factor analysis: 9 factors<br><br>Cronbach's $\alpha > .70$ (for subscales)
**Grey Literature**				
MacColl Center for Health Care Innovation 2014<br><br>Google Scholar citations: 0<br><br>Article quality rating: N/A	Patient-Centered Medical Home Assessment (PCMH-A)	36-item self-assessment tool to give clinical practices a method for gauging progress in the medical home implementation process:<br>• 8 subscales: 1) engaged leadership, 2) quality improvement strategy, 3)empanelment, 4) continuous & team-based healing relationships, 5) organized, evidence-based care, 6) patient-centered interactions, 7) enhanced access, and 8) care coordination	USA<br><br>n = 65 sites were used to extensively test the measure	Not reported

Author	Name	Description	Setting & Sample	Psychometrics
Martin et al. 2007<br><br>Google Scholar citations: 40<br><br>Article quality rating: N/A	Whole System Measures (WSM)	13-indicators, as a part of a balanced set of measures, designed to measure quality across the continuum of care:<br>• 1) rate of adverse events, 2) incidence of nonfatal occupational injuries and illnesses, 3) hospital standardized mortality ratio effective, 4) unadjusted raw mortality percentage, 5) functional health outcomes score, 6) hospital readmission percentage, 7) reliability of core measures, 8) patient satisfaction with care score, 9) patient experience score, 10) days to third next available appointment, 11) hospital days per decedent during the last six months of life, 12) health care cost per capita, and 13) equity (stratification of WSM)<br>• The WSM offers recommended measurement methods for each of the 13 indicators<br><br>The 13 indicators align with the Institute of Medicine's 6-dimensions of quality care that services are safe, effective, patient-centered, timely, efficient, and equitable.	Europe (United Kingdom, Sweden) and USA<br><br>n = 10 individuals met to discuss measures of health system quality<br><br>n = 30 health systems tested the original WSM	Each indicator's measure was tested individually

Author	Name	Description	Setting & Sample	Psychometrics
SAMHSA-HRSA Center for Integrated Health Solutions<br>Google Scholar citations: 0<br>Article quality rating: N/A	Organizational Assessment Toolkit for Primary and Behavioural Health Care Integration (OATI)	This toolkit includes 4 tools to help organizations to plan, prepare, and assess their steps towards providing services that are more integrated:<br>• 1) The Partnership Checklist<br>• 2) The Executive Walkthrough<br>• 3) The Administrative Readiness Tool (ART) for Primary Health Behavioral Integration<br>• 4) The COMPASS-Primary Health and Behavioral Health™	Not reported	Not reported

Note: Google Scholar citations as of January 24, 2017
